# ﻿Morphological and molecular analyses revealed four new wood-inhabiting fungal species (Hymenochaetales, Basidiomycota) from Yunnan

**DOI:** 10.3897/mycokeys.117.146236

**Published:** 2025-04-30

**Authors:** Yinglian Deng, Meng Chen, Linfeng Liu, Qizhen Li, Sicheng Zhang, Haisheng Yuan, Changlin Zhao

**Affiliations:** 1 Yunnan Provincial Key Laboratory for Conservation and Utilization of In-forest Resource, the Key Laboratory of Forest Resources Conservation and Utilization in the Southwest Mountains of China Ministry of Education, Southwest Forestry University, Kunming 650224, China; 2 College of Forestry, Southwest Forestry University, Kunming 650224, China; 3 Key Laboratory of Forest Ecology and Management, Institute of Applied Ecology, Chinese Academy of Sciences, Shenyang 110016, China

**Keywords:** Biodiversity, new taxa, phylogenetic analyses, Southwestern China, taxonomy

## Abstract

Hymenochaetales is one of the fungal orders mainly composed of wood-inhabiting macrofungi within the class Agaricomycetes, Basidiomycota. Four new Hymenochaetales wood-inhabiting fungi, *Hymenochaetebannaensis*, *Lyomycesasiaticus*, *Peniophorellaalbohymenia*, and *P.punctata* collected from China are proposed based on morphological characteristics and molecular evidence. *H.bannaensis* is distinguished by flocculent basidiomata with cinnamon to yellowish brown to rust-brown hymenial surface, generative hyphae with simple septa and broadly ellipsoid to globose basidiospores. *L.asiaticus* is characterized by the membranaceous basidiomata with white to cream hymenial surface with tuberculate, a monomitic hyphal system with clamped generative hyphae and ellipsoid basidiospores. In addition, *P.albohymenia* is delimited by membranaceous basidiomata with white hymenial surface, four types of cystidia as stephanocyst, fusiform, cylindrical and capitate, and ellipsoid basidiospores. *P.punctata* is unique in the membranaceous, punctate basidiomata with white to pale yellow hymenial surface, fusiform cystidia, and allantoid basidiospores. Sequences of ITS and nLSU rRNA markers based on phylogenetic analyses were performed using the Maximum Likelihood, Maximum Parsimony and Bayesian Inference methods.

## ﻿Introduction

In terms of taxonomic status, wood inhabiting fungi mainly refer to the classification within the class Agaricomycetes, Basidiomycota (James et al. 2020; Dai et al. 2021; Wijayawardene et al. 2022; Yuan et al. 2023; Zhao et al. 2024; Dai et al. 2025). Most of basidiomycetes are white-rot fungi, which are responsible for degradation of the lignin in the forest ecosystems (Floudas et al. 2012; Osman and El-Nuby 2023). Rot type can be inferred from direct examination of wood substrates or from cultural tests, in which it was classified to three groups, brown rot, soft rot, and white rot fungi, and all species of Hymenochaetales cause white rot (Justo et al. 2017; Wang et al. 2023a).

Hymenochaetales is one of the fungal orders being mainly composed of wood-inhabiting fungi within Agaricomycetes, Basidiomycota (Wang et al. 2023b; Zhou et al. 2023; Dai et al. 2025). This order includes fascinating basidiomycete fungi that occupies a distinct ecological niche, in which there are many variations of the basidiomata types within Hymenochaetales (Larsson et al. 2006; Wu et al. 2019a; Guan et al. 2023). Hymenochaetales comprises a number of representative wood-inhabiting fungal taxa, which includes diverse hymenophoral morphologies as hydnoid, corticioid, and polyporoid (Zhao et al. 2023a; Yuan et al. 2024). Currently, 14 families, 83 genera, and 1205 species, are accepted in Hymenochaetales, and include wood-inhabiting and ectomycorrhizal fungi (Hibbett et al. 2014; Wang et al. 2023a; He et al. 2024). However, the species diversity has been well explored worldwide, therefore more new taxa need to be discovered. Furthermore, the order has various morphological characters for basidiomata, in which all characteristics are also found in the other orders (Polyporales, Russulales) in Agaricomycetes (Wu and Yuan 2020).

On the basis of the frequent inclusion of data from DNA sequences in many phylogenetic studies, the classification of the wood-inhabiting fungi has been updated continuously (Yurchenko et al. 2020a; Mao et al. 2023; Zhang et al. 2023b). Wang et al. (2023a) implemented a multi-locus phylogenetic analysis on the basis of nSSU, ITS, nLSU, mt-SSU, *tef1α*, *rpb1*, and *rpb2* genes and updated the taxonomic framework of Hymenochaetales. The results have shown that 45 genera were accepted in Hymenochaetaceae, including the genus *Hymenochaete* (Wang et al. 2023a), and Hymenochaetaceae comprised three major genera *Phellinus* sensu lato, *Hymenochaete* and *Inonotus* sensu lato (Larsen and Cobb-Poulle 1990; Léger 1998; Ryvarden 2005; Wu et al. 2022a). The results showed that the genus *Hymenochaete* (Hymenochaetaceae) was closely grouped with *Hymenochaetopsis* S.H. He & Jiao Yang, and they were almost indistinguishable in morphology (Larsson et al. 2006; He and Dai 2012; Baltazar et al. 2014; Parmasto et al. 2014; Yang et al. 2016). According to the anatomical features, the genus has been divided into four sections, *Hymenochaete*, *Fultochaete* Escobar ex Léger, *Gymnochaete* Escobar ex Léger and *Paragymnochaete* Léger (Léger 1998; He and Dai 2012).

Molecular studies of *Hyphoderma* Wallr. revealed that the reinstatement of *Peniophorella* resulted in 19 new combinations in the genus *Peniophorella* and all species nested in the hymenochaetoid clade (Larsson 2007b). Miettinen et al. (2019) revealed that *P.praetermissa* (P. Karst.) K.H. Larss. and *P.tsugae* (Burt) K.H. Larss. grouped together and clustered in Hymenochaetales. Telleria et al. (2012) studied *Hyphoderma* and its discrimination from closely related taxa and indicated that eight *Peniophorella* taxa were closely grouped and distinct from *Hyphoderma* s.s. Justo et al. (2017) revised the family-level classification of the order Polyporales (Basidiomycota) and proposed that 20 species were classified in *Peniophorella* located in the family Hyphodermataceae (Hymenochaetales). Morphological studies and phylogenetic analyses reported six new taxa of *Peniophorella* and displayed the taxonomic status for the new taxa within the genus (Guan et al. 2020; Xu et al. 2020; Yurchenko et al. 2020a). Wang et al. (2023a) proposed that the genus *Peniophorella* bears affinity to a new family Peniophorellaceae.

Due to the lack of sequences of some wood-inhabiting fungal taxa, it is difficult to clearly distinguish many genera in Schizoporaceae, therefore, a broad concept of *Hyphodontia* s.l. was accepted (Yurchenko and Wu 2014; Riebesehl et al. 2015; Riebesehl and Langer 2017). Based on the nuclear DNA sequence data, six well-distinguished clades as the *Hastodontia* clade, the *Hyphodontia* clade, the *Lagarobasidium* clade, the *Kneiffiella*-*Alutaceodontia* clade, the *Xylodon*-*Lyomyces*-*Rogersella* clade and the *Xylodon*-*Schizopora*-*Palifer* clade, were included (Yurchenko and Wu 2014). Inferred from the phylogenetical studies for *Hyphodontia* s.l., the genus *Lyomyces* was nested within the *Xylodon-Lyomyces-Rogersella* clade (Yurchenko and Wu 2014). Riebesehl et al. (2019) clarified the generic concept and their phylogenetic reconstruction of *Lyomyces* and the species *L.sambuci* (Pers.) P. Karst. was sister to *L.crustosus* (Pers.) P. Karst (Riebesehl et al. 2019).

In this study, four taxa from three genera of Hymenochaetales were collected from Yunnan, China and the phylogenetic relationships are studied based on ITS-nLSU gene regions. Therefore, exploring the diversity and taxonomic status of these new taxa using different characters will be significant for wood-inhabiting fungi in Yunnan. Morphology and phylogenetic analyses reveal that these are new to science.

## ﻿Materials and methods

### ﻿Sample collection and morphology examination

Fresh basidiomata of the fungi were collected from Xishuangbanna and Zhaotong of Yunnan Province, China, from September 2023 to January 2024. Specimens were dried in an electric food dehydrator at 40 °C, then sealed and stored in an envelope bag and deposited in the herbarium of the Southwest Forestry University (SWFC), Kunming, Yunnan Province, China. Macromorphological descriptions are based on field notes and photos captured in the field and lab. Color terminology was followed as mentioned by Petersen (1996). Micromorphological data were obtained from the dried specimens when observed under a light microscope (Nikon Ni, Tokyo, Japan) following the previous study (Cui et al. 2019; Zhao et al. 2023b; Dong et al. 2024). The following abbreviations are used: KOH = 5% potassium hydroxide water solution, CB = Cotton Blue, CB– = acyanophilous, IKI = Melzer’s Reagent, IKI– = both inamyloid and indextrinoid, L = mean spore length (arithmetic average for measured spores), W = mean spore width (arithmetic average for measured spores), Q = variation in the L/W ratios between the specimens studied and n = a/b (number of spores (a) measured from given number (b) of specimens). Standardized sampling of microstructures for measurement includes basidiospores (30), basidia, basidioles and cystidia (5), setae and hyphae diameter (10) (Cui et al. 2019; Zhao et al. 2023b; Dong et al. 2024).

### ﻿Molecular phylogeny

The EZNA HP Fungal DNA Kit (Omega Biotechnologies Co., Ltd., Kunming, China) was used to extract DNA with some modifications from the dried specimens. The nuclear ribosomal ITS region was amplified with primers ITS5 and ITS4 (White et al. 1990). The PCR procedure for ITS was as follows: initial denaturation at 95 °C for 3 min, followed by 35 cycles at 94 °C for 40 s, 58 °C for 45 s and 72 °C for 1 min, and a final extension of 72 °C for 10 min. The nuclear nLSU region was amplified with primer pair LR0R and LR7 (Vilgalys and Hester 1990; Rehner and Samuels 1994). The PCR procedure for nLSU was as follows: initial denaturation at 94 °C for 1 min, followed by 35 cycles at 94 °C for 30 s, 48 °C for 1 min and 72 °C for 1.5 min, and a final extension of 72 °C for 10 min. The PCR procedure for ITS and nLSU followed previous study (Zhao and Wu 2017). All of the newly generated sequences were deposited in NCBI GenBank (https://www.ncbi.nlm.nih.gov/genbank/) (Table 1).

**Table 1.** List of species, specimens and GenBank accession numbers of sequences used in this study. [* Indicates type materials].

Species name	Sample no.	GenBank accessions no.		References
		ITS	nLSU	
*Basidioradulummayi*	LWZ 20180510-18	MN017785	MN017792	Wang et al. (2023a)
*B.radula*	LWZ 20201017-62	ON063684	ON063884	Wang et al. (2023a)
*Boletopsisleucomelaena*	PBM2678	DQ484064	DQ154112	Liu et al. (2022)
*Fasciodontiabrasiliensis*	MSK-F 7245a	MK575201	MK598734	Yurchenko et al. (2020a)
*F.bugellensis*	KAS-FD 10705a	MK575203	MK598735	Yurchenko et al. (2020a)
*F.bannaensis*	MUCL 46950	GU461943	EF429218	Nie et al. (2017)
*F.gabonensis*	MUCL 47576	GU461971	GU461990	Wang et al. (2023a)
*F.punctate*	MUCL 47629	GU461950	GU461982	Nie et al. (2017)
*F.rhamnoides*	LWZ 20180905-15	ON063643	ON063842	Wang et al. (2023a)
*Hydnoporiapinicola*	CLZhao 22505	OR094493	OR449924	Dong et al. (2024)
*H.pinicola*	CLZhao 27154	OR094494	OR449925	Dong et al. (2024)
*H.yunnanensis*	CLZhao 6123 *	OR094498	OR449929	Dong et al. (2024)
*H.yunnanensis*	CLZhao 6227	OR094499	OR449930	Dong et al. (2024)
*Hymenochaeteacerosa*	He 338	JQ279543	JQ279657	He et al. (2017)
*H.adusta*	He 207	JQ279523	KU975497	He et al. (2017)
*H.angustispora*	Dai 17045	MF370592	MF370598	He et al. (2017)
*H.angustispora*	Dai 17049	MF370593	MF370599	He et al. (2017)
*H.anomala*	He 592	JQ279566	JQ279650	He et al. (2017)
*H.asetosa*	Dai 10756	JQ279559	JQ279642	He et al. (2017)
*H.attenuate*	He 28	JQ279526	JQ279633	He et al. (2017)
*H.bambusicola*	He 4116	KY425674	KY425681	He et al. (2017)
*H.berteroi*	He 1488	KU975459	KU975498	He et al. (2017)
*H.biformisetosa*	He 1445	KF908247	KU975499	Yang and He (2014)
*H.boddingii*	MEH 66068	MN030343	MN030345	Du et al. (2021)
*H.boddingii*	MEH 69996	MN030341	MN030347	Du et al. (2021)
*H.borbonica*	CBS 731.86	MH862026	MH873716	Du et al. (2021)
*H.cana*	He 1305	KF438169	KF438172	He et al. (2017)
*H.cinnamomea*	He 755	JQ279548	JQ279658	He et al. (2017)
*H.colliculosa*	Dai 16427	MF370595	MF370602	He et al. (2017)
*H.colliculosa*	Dai 16428	MF370596	MF370603	He et al. (2017)
*H.conchata*	MEH 70144	MF373838	—	Du et al. (2021)
*H.contiformis*	He 1166	KU975461	KU975501	He et al. (2017)
*H.cruenta*	He 766	JQ279595	JQ279681	He et al. (2017)
*H.cyclolamellata*	Cui 7393	JQ279513	JQ279629	He et al. (2017)
*H.damicornis*	URM 84261	KC348466	—	Du et al. (2021)
*H.damicornis*	URM 84263	KC348467	—	Du et al. (2021)
*H.denticulata*	He 1271	KF438171	KF438174	He et al. (2017)
*H.dracaenicola*	Dai 22090	MW559797	MW559802	Du et al. (2021)
*H.dracaenicola*	Dai 22096	MW559798	MW559803	Du et al. (2021)
*H.duportii*	AFTOL ID666	DQ404386	AY635770	He et al. (2017)
*H.epichlora*	He 525	JQ279549	JQ279659	He and Dai (2012)
*H.floridea*	He 536	JQ279597	JQ279683	He and Dai (2012)
*H.fuliginosa*	He 1188	KU975465	KU975506	Du et al. (2021)
*H.fulva*	He 640	JQ279565	JQ279648	He and Dai (2012)
*H.globispora*	He 911	—	KU975508	Du et al. (2021)
*H.huangshanensis*	He 432	JQ279533	JQ279671	He and Dai (2012)
*H.innexa*	He 555	JQ279584	JQ279674	He and Dai (2012)
*H.japonica*	He 245	JQ279590	JQ279680	He and Dai (2012)
*H.legeri*	He 960	KU975469	KU975511	He et al. (2017)
*H.longispora*	He 217	JQ279537	KU975514	He and Dai (2012)
*H.luteobadia*	He 8	JQ279569	KU975515	He and Dai (2012)
*H.macrochloae*	ARAN-fungi 7079	MF990738	MF990743	Du et al. (2021)
*H.megaspora*	He 302	JQ279553	JQ279660	He and Dai (2012)
*H.minor*	He 933	JQ279555	JQ279654	He and Dai (2012)
*H.minuscula*	He 253	JQ279546	KU975516	He and Dai (2012)
*H.murina*	He 569	JQ716406	JQ716412	He et al. (2017)
*H.muroiana*	He 405	JQ279542	KU975517	Du et al. (2021)
*H.nanospora*	He 475	JQ279531	JQ279672	He and Dai (2012)
*H.ochromarginata*	He 47	JQ279579	JQ279666	He and Dai (2012)
*H.orientalis*	He 4601	KY425677	KY425685	He et al. (2017)
*H.parmastoi*	He 277	JQ780064	—	He et al. (2017)
*H.paucisetigera*	Cui 7845	JQ279560	JQ279644	He and Dai (2012)
*H.quercicola*	He 373	KU975474	KU975521	He et al. (2017)
*H.rhabarbarina*	He 280	JQ279574	KY425688	He and Dai 2012
*H.rheicolor*	Cui 8317	JQ279529	—	Du et al. (2021)
*H.rhododendricola*	He 389	JQ279577	JQ279653	He and Dai (2012)
*H.rubiginosa*	He 1049	JQ716407	JQ279667	Yang et al. (2016)
*H.rubiginosa*	LWZ 20201017-32	ON063655	—	Wang et al. (2023a)
*H.rufomarginata*	He 1489	KU975477	KU975524	He et al. (2017)
*H.separabilis*	He 460	JQ279572	JQ279655	He and Dai (2012)
*H.setipora*	Cui 6301	JQ279515	JQ279639	Du et al. (2021)
*H.sharmae*	66088	MK588753	MK588836	Du et al. (2021)
*H.sharmae*	CAL 1535	KY929017	KY929018	Du et al. (2021)
*H.sinensis*	CLZhao 26040	OR659001	PP425893	Li et al. (2024)
*H.spathulata*	He 685	JQ279591	KU975529	He et al. (2017)
*H.sphaericola*	He 303	JQ279599	JQ279684	Li et al. (2024)
*H.sphaericola*	LWZ 20190808-2b	ON063656	ON063855	Li et al. (2024)
*H.sphaerospora*	He 715	JQ279594	KU975531	He et al. (2017)
*H.subferruginea*	Cui 8122	JQ279521	—	Du et al. (2021)
*H.subferruginea*	He 1598	KU975481	—	Du et al. (2021)
*H.tabacina*	Dai 11635	JQ279563	JQ279647	He and Dai (2012)
*H.tasmanica*	He 449	JQ279582	JQ279663	He et al. (2017)
*H.tenuis*	He 779	JQ279538	JQ279641	Du et al. (2021)
*H.tongbiguanensis*	He 1552	KF908248	KU975532	He et al. (2017)
*H.tropica*	He 574	JQ279587	JQ279675	He et al. (2017)
*H.ulmicola*	He 864	JQ780065	KU975534	He et al. (2017)
*H.unicolor*	He 468a	JQ279551	JQ279662	He et al. (2017)
*H.verruculosa*	Dai 17052	MF370594	MF370601	He et al. (2017)
*H.villosa*	He 537	JQ279528	JQ279634	He et al. (2017)
*H.xerantica*	Cui 9209	JQ279519	JQ279635	Du et al. (2021)
*H.xerantica*	LWZ 20190814-13b	ON063657	ON063856	Wang et al. (2023a)
***H.bannaensis***	**CLZhao 35721** *	**PQ847494**	**PQ847499**	**Present study**
***H.bannaensis***	**CLZhao 35884**	**PQ847495**	**PQ847500**	**Present study**
*H.yunnanensis*	He 1447	KU975486	KU975538	He et al. (2017)
*Hyphodontiaarguta*	KHL 11938 (GB)	EU118632	EU118633	Larsson (2007a)
*H.arguta*	LWZ 20180905-6	ON063672	ON063871	Wang et al. (2023a)
*H.borbonica*	FR-0219441	KR349240	NG_068856	Riebesehl et al. (2015)
*H.pachyspora*	LWZ20170908-5	MT319426	MT319160	Wang et al. (2023a)
*H.pachyspora*	LWZ20180905-6	MT319425	MT319159	Wang et al. (2021)
*H.pallidula*	He6087	OM100749	OM083983	Unpublished
*H.pallidula*	He6092	OM100750	OM083984	Unpublished
*H.wongiae*	LWZ20180414-16	MT319414	MT319146	Wang et al. (2023a)
*H.wongiae*	LWZ20180417-16	MT319415	MT319147	Wang et al. (2023a)
*Lyomycesalbopulverulentus*	CLZhao 21478	OP730712	OP730724	Guan et al. (2023)
*L.allantosporus*	FR-0249548	KY800397	KY795963	Yurchenko et al. (2017)
*L.allantosporus*	KAS-GEL4933	KY800401	KY795965	Yurchenko et al. (2017)
***L.asiaticus***	**CLZhao 35703**	**PQ847492**	**PQ847501**	**Present study**
***L.asiaticus***	**CLZhao 35719** *	**PQ847493**	**PQ847502**	**Present study**
*L.bambusinus*	CLZhao 4808	MN945970	—	Chen and Zhao (2020)
*L.bambusinus*	CLZhao 4831	MN945968	MW264919	Chen and Zhao (2020)
*L.cremeus*	CLZhao 4138	MN945974	MW264922	Chen and Zhao (2020)
*L.cremeus*	CLZhao 8295	MN945972	—	Chen and Zhao (2020)
*L.crustosus*	TASM:YG G39	MF382993	—	Gafforov et al. (2017)
*L.crustosus*	UC2022841	KP814310	—	Guan et al. (2023)
*L.densiusculus*	Ryvarden 44818	OK273853	OK273853	Viner et al. (2022)
*L.elaeidicola*	LWZ20180411-19	MT319457	MT319190	Wang et al. (2021)
*L.elaeidicola*	LWZ20180411-20	MT319458	NG_153910	Wang et al. (2021)
*L.erastii*	23cSAMHYP	JX857800	—	Unpublished
*L.erastii*	TASM:YG 022	MF382992	—	Gafforov et al. (2017)
*L.fimbriatus*	Wu910620-7	MK575209	—	Yurchenko et al. (2020a)
*L.fimbriatus*	Wu911204-4	MK575210	MK598740	Yurchenko et al. (2020a)
*L.fissuratus*	CLZhao 4291	MW713738	MW713730	Luo et al. (2021b)
*L.fissuratus*	CLZhao 4352	MW713742	MW713732	Luo et al. (2021b)
*L.fumosus*	CLZhao 8188	MW713744	MW713736	Luo et al. (2021b)
*L.gatesiae*	LWZ20180515-3	MT319447	MT319181	Wang et al. (2021)
*L.gatesiae*	LWZ20180515-32	MT319448	MT319182	Wang et al. (2021)
*L.griseliniae*	KHL 12971 (GB)	DQ873651	DQ873651	Larsson et al. (2006)
*L.hengduanensis*	CLZhao 20627	OR793233	PP657611	Yuan et al. (2024)
*L.hengduanensis*	CLZhao 25551	OR658999	PP657610	Yuan et al. (2024)
*L.incanus*	CLZhao 22813	OR094480	OR449935	Dong et al. (2024)
*L.incanus*	CLZhao 22900 *	OR094481	OR449936	Dong et al. (2024)
*L.juniper*	FR-0261086	KY081799	—	Riebesehl and Langer (2017)
*L.macrosporus*	CLZhao 4516	MN945977	MW264920	Chen and Zhao (2020)
*L.mascarensis*	KAS-GEL4833	KY800399	KY795964	Yurchenko et al. (2020a)
*L.mascarensis*	KAS-GEL4908	KY800400	—	Yurchenko et al. (2020a)
*L.microfasciculatus*	CLZhao 5109	MN954311	MW264921	Chen and Zhao (2020)
*L.niveomarginatus*	CLZhao 16360	PP537949	PP657607	Yuan et al. (2024)
*L.niveus*	CLZhao 6431	MZ262541	MZ262526	Luo et al. (2021b)
*L.niveus*	CLZhao 6442	MZ262542	MZ262527	Luo et al. (2021b)
*L.ochraceoalbus*	CLZhao 4385	MZ262535	MZ262521	Luo et al. (2021b)
*L.ochraceoalbus*	CLZhao 4725	MZ262536	MZ262522	Luo et al. (2021b)
*L.orientalis*	GEL3376	DQ340325	DQ340351	Yurchenko et al. (2017)
*L.pruni*	GEL2327	DQ340312	DQ340349	Larsson et al. (2006)
*L.pruni*	Ryberg 021018 (GB)	DQ873624	—	Larsson et al. (2006)
*L.sambuci*	83SAMHYP	JX857721	—	Yurchenko et al. (2017)
*L.sambuci*	KAS-JR7	KY800402	KY795966	Yurchenko et al. (2017)
*L.vietnamensis*	He 3260	MW507086	MW507028	Yurchenko et al. (2017)
*L.wuliangshanensis*	He 3498	MW507087	MW507029	Chen and Zhao (2020)
*L.wuliangshanensis*	He 4765	MW507090	MW507032	Chen and Zhao (2020)
*L.wumengshanensis*	CLZhao 29374	OR803021	PP657613	Yuan et al. (2024)
*L.wumengshanensis*	CLZhao 32800	OR899211	PP657614	Yuan et al. (2024)
*L.yunnanensis*	CLZhao 2463	OP730711	OP730723	Guan et al. (2023)
*L.yunnanensis*	CLZhao 9375	OP730710	—	Guan et al. (2023)
*L.zhaotongensis*	CLZhao 32878	PP537950	PP657609	Yuan et al. (2024)
*Nigrofomesmelanoporus*	JV 1704/39	MF629835	MF629831	Wang et al. (2023a)
*N.sinomelanoporus*	Cui 5277	MF629836	MF629832	Wang et al. (2023a)
***Peniophorellaalbohymenia***	**CLZhao 33187** *	**PQ811412**	**PQ847496**	**Present study**
***P.albohymenia***	**CLZhao 33257**	**PQ811413**	—	**Present study**
*P.aspersa*	TNM F24809	MN062097	MN062142	Yurchenko et al. (2020b)
*P.aspersa*	TNM F32708	MN062099	MN062144	Yurchenko et al. (2020b)
*P.cremea*	CLZhao 1606	MT955162	—	Xu et al. (2020)
*P.cremea*	CLZhao 1719	MT955163	—	Xu et al. (2020)
*P.crystallifera*	LWZ 20210626-4a	ON063685	ON063885	Wang et al. (2023a)
*P.crystallifera*	TNM F30331	MN062100	MN062147	Yurchenko et al. (2020b)
*P.daweishanensis*	CLZhao 18600 *	OR094501	OR449932	Dong et al. (2024)
*P.echinocystis*	KHL 6284	DQ677494	DQ681200	Larsson (2007b)
*P.fissurata*	CLZhao 5848	MN864262	OM985777	Guan et al. (2020)
*P.fissurata*	CLZhao 9421	MN864260	OM985776	Guan et al. (2020)
*P.guttulifera*	CBS 107303	LT603016	LT603001	Kolařík and Vohník (2018)
*P.guttulifera*	NH 12012 (GB)	DQ647501	—	Hallenberg et al. (2007)
*P.odontiiformis*	TMI 21347	DQ647496	—	Hallenberg et al. (2007)
*P.odontiiformis*	TMI 6824	DQ647500	—	Hallenberg et al. (2007)
*P.olivacea*	CLZhao 25896 *	OR094502	OR449933	Dong et al. (2024)
*P.pallida*	UC 2022844	KP814208	—	Rosenthal et al (2017)
*P.pallida*	UC 2022887	KP814201	—	Rosenthal et al (2017)
*P.pertenuis*	NH 12429 (GB)	DQ647486	—	Hallenberg et al. (2007)
*P.pertenuis*	NH 15115 (GB)	DQ647487	—	Hallenberg et al. (2007)
*P.praetermissa*	NH 10986 (GB)	DQ647462	—	Hallenberg et al. (2007)
*P.praetermissa*	NH 11192 (GB)	DQ647461	—	Hallenberg et al. (2007)
*P.pubera*	CBS:464.86	MH861988	MH873680	Vu et al. (2019)
*P.pubera*	LWZ 20210624-16b	ON063687	ON063887	Wang et al. (2023a)
***P.punctata***	**CLZhao 33720**	**PQ811414**	**PQ847497**	**Present study**
***P.punctata***	**CLZhao 33732** *	**PQ811415**	**PQ847498**	**Present study**
*P.reticulate*	CLZhao 17066	OM985746	OM985783	Dong et al. (2024)
*P.reticulate*	TNM F22559	MN062103	MN062151	Yurchenko et al. (2020b)
*P.rude*	LWZ 20171026-7	ON063688	ON063888	Wang et al. (2023a)
*P.subpraetermissa*	LWZ 20190816-3b	ON063689	ON063889	Wang et al. (2023a)
*P.subpraetermissa*	Wu 950627	DQ647493	—	Hallenberg et al. (2007)
*P.yunnanensis*	CLZhao 4810	MN864263	OM985788	Guan et al. (2020)
*P.yunnanensis*	CLZhao 6137	MN864266	—	Guan et al. (2020)
*Phylloporiaoreophila*	LWZ 20190811-27a	ON063665	ON063865	Wang et al. (2023a)
*P.radiate*	LWZ 20141122-6	ON063666	ON063866	Wang et al. (2023a)
*Porodaedaleahimalayensis*	LWZ 20180903-21	ON063667	ON063867	Wang et al. (2023a)
*P.laricis*	LWZ 20190724-9	ON063668	ON063868	Wang et al. (2023a)
*Sanghuangporusquercicola*	LWZ 20170821-18	ON063669	ON063869	Wang et al. (2023a)
*S.weigelae*	LWZ 20210623-2a	ON063671	ON063870	Wang et al. (2023a)
*Skvortzoviadabieshanensis*	LWZ 20201017-55	MW414514	MW414460	Wang et al. (2023a)
*S.dabieshanensis*	LWZ 20210918-15b	ON063694	ON063894	Wang et al. (2023a)
*S.furfurella*	KHL 10180	DQ873649	DQ873649	Yu et al. (2021)
*S.incana*	CLZhao 16338 *	OR096179	OR449950	Dong et al. (2024)
*S.yunnanensis*	CLZhao 16084	MW472754	MW473473	Wang et al. (2023a)
*S.yunnanensis*	CLZhao 16181	MW472755	MW473474	Dong et al. (2021)
*Thelephoraganbajun*	ZRL20151295	LT716082	KY418908	Liu et al. (2022)
*Tubulicrinisglebulosus*	LWZ 20180903-13	ON063705	—	Wang et al. (2023a)
*T.pini*	CLZhao 3679	—	OR449951	Dong et al. (2024)
*T.pini*	CLZhao 6881 *	OR096210	OR449952	Dong et al. (2024)
*T.yunnanensis*	CLZhao 3418	MT153879	MT153886	He et al. (2020)
*T.yunnanensis*	CLZhao 9717	MT153880	MT153887	He et al. (2020)
*Xylodonolivaceobubalinus*	CLZhao 25164	OR167771	OR449947	Dong et al. (2024)
*X.olivaceobubalinus*	CLZhao 25174 *	OR167772	OR449948	Dong et al. (2024)

**Figure 1.**
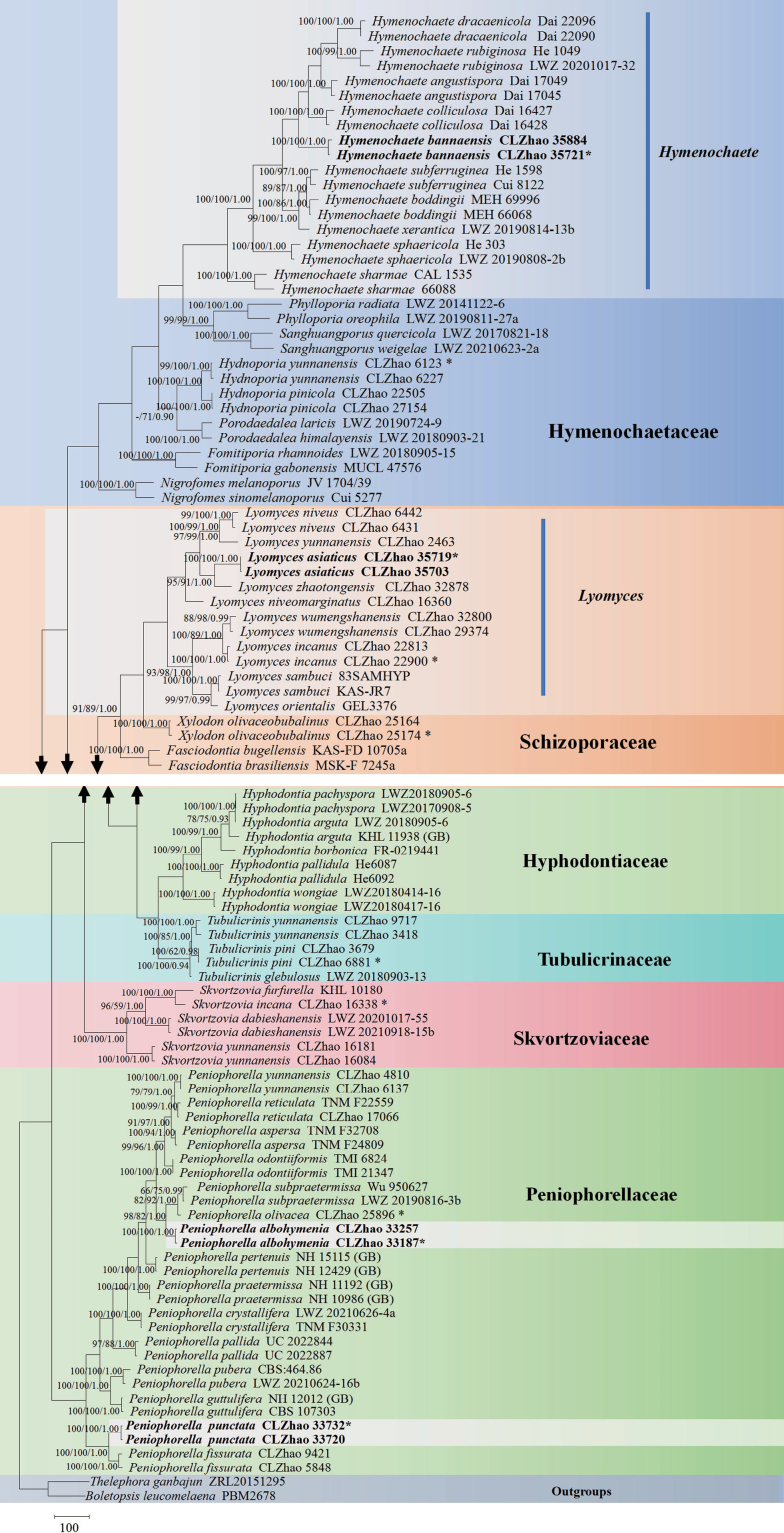
Maximum Parsimony strict consensus tree illustrating the phylogeny of the order Hymenochaetales based on ITS+nLSU sequences. Branches are labeled with Maximum Likelihood bootstrap values ≥ 70%, parsimony bootstrap values ≥ 50% and Bayesian posterior probabilities ≥ 0.95, respectively.

**Figure 2.**
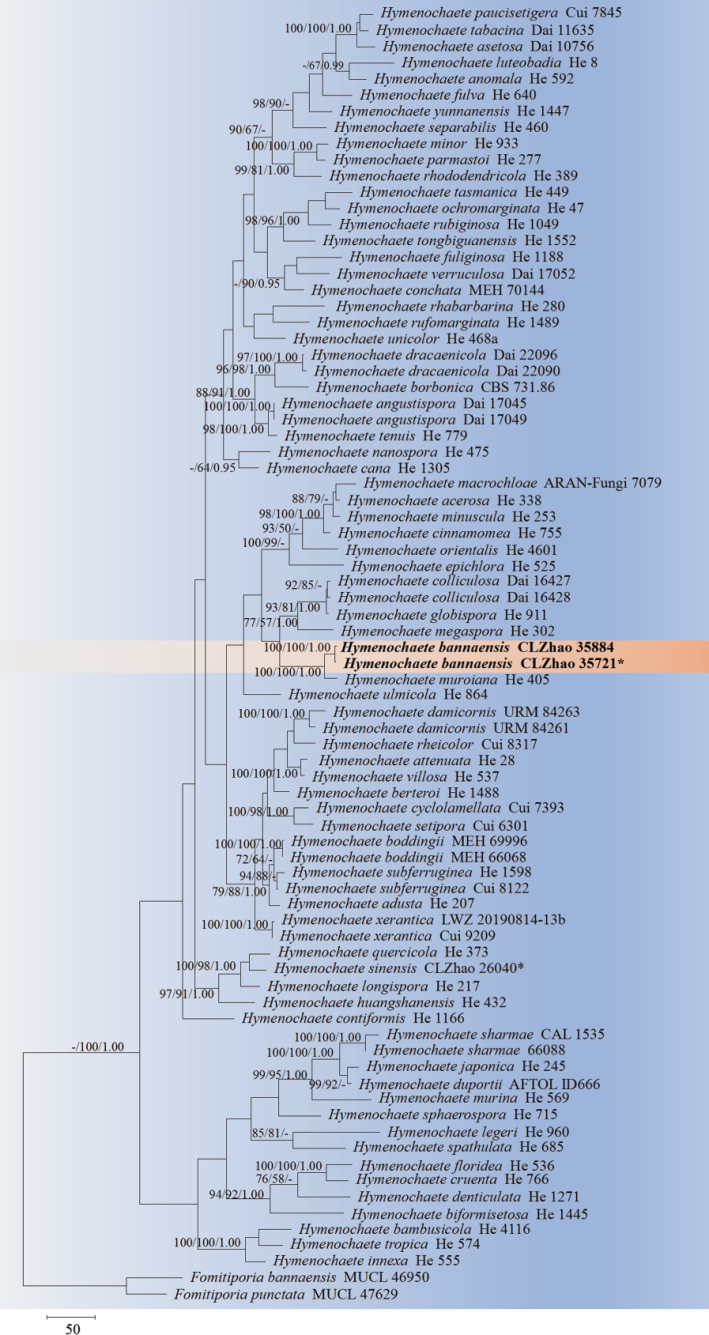
Maximum Parsimony strict consensus tree illustrating the phylogeny of the two new species and related species in *Hymenochaete*, based on ITS+nLSU sequences. Branches are labeled with Maximum Likelihood bootstrap values ≥ 70%, parsimony bootstrap values ≥ 50% and Bayesian posterior probabilities ≥ 0.95, respectively.

**Figure 3.**
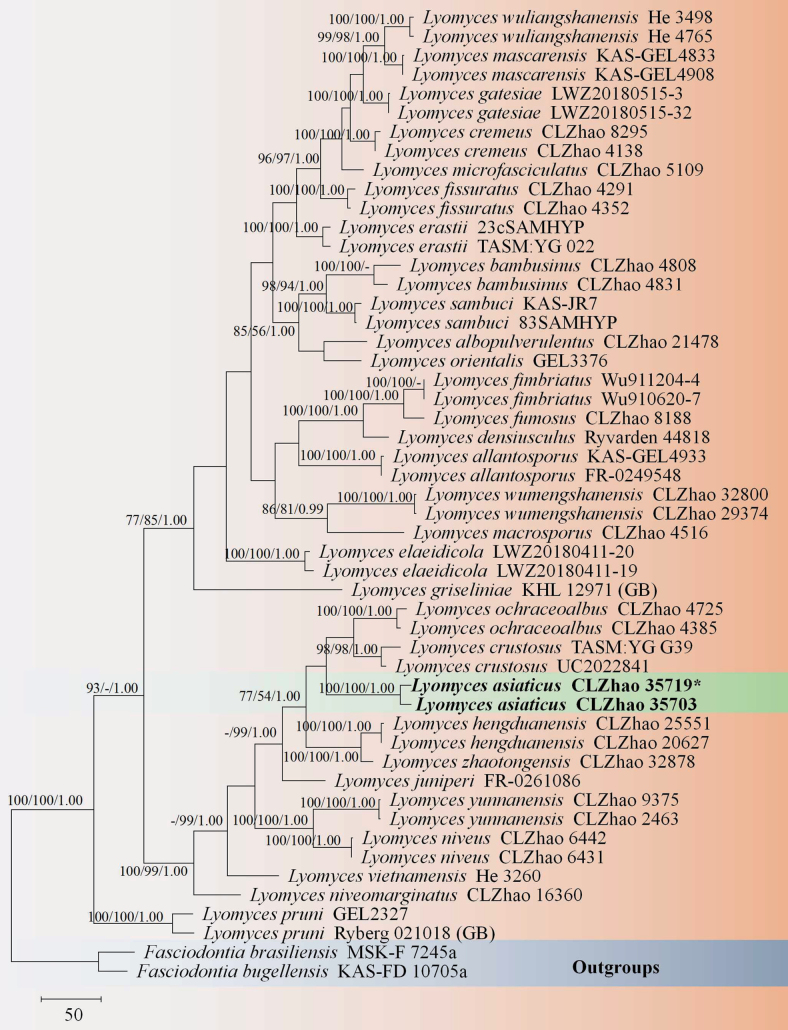
Maximum Parsimony strict consensus tree illustrating the phylogeny of the two new species and related species in *Lyomyces*, based on ITS+nLSU sequences. Branches are labeled with Maximum Likelihood bootstrap values ≥ 70%, parsimony bootstrap values ≥ 50% and Bayesian posterior probabilities ≥ 0.95, respectively.

**Figure 4.**
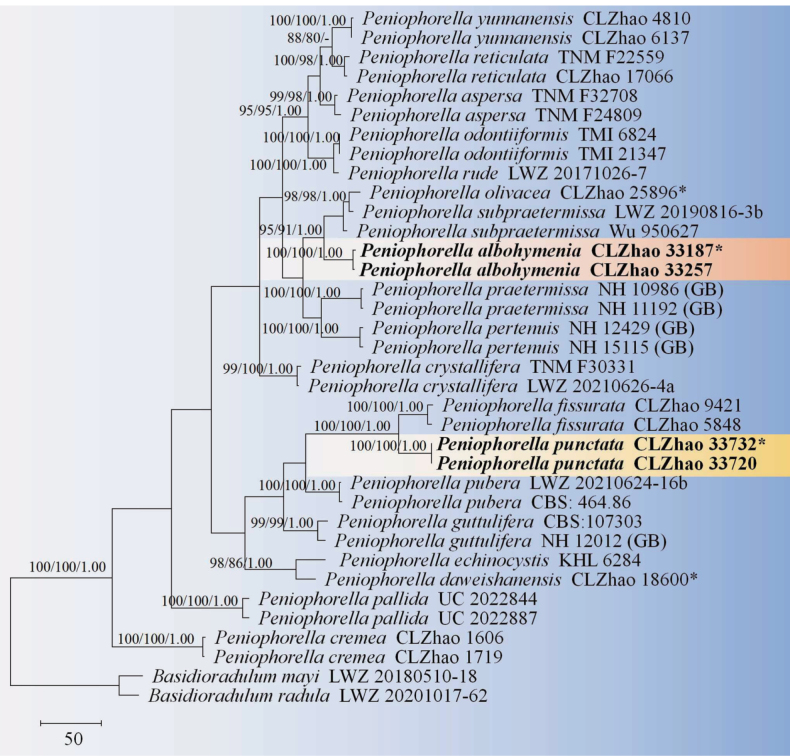
Maximum Parsimony strict consensus tree illustrating the phylogeny of the two new species and related species in *Peniophorella*, based on ITS+nLSU sequences. Branches are labeled with Maximum Likelihood bootstrap values ≥ 70%, parsimony bootstrap values ≥ 50% and Bayesian posterior probabilities ≥ 0.95, respectively.

The sequences were aligned in MAFFT version 7 (Katoh et al. 2019) using the G-INS-i strategy. The alignment was adjusted manually using AliView version 1.27 (Larsson 2014). The combined dataset was deposited in TreeBASE (https://treebase.org/treebase-web/home.html; submission ID 32063). Sequences of *Boletopsisleucomelaena* (Pers.) Fayod and *Thelephoraganbajun* M. Zang, retrieved from GenBank were used as the outgroup in the ITS+nLSU analysis (Fig. 1; Dong et al. 2024). Sequences of *Fomitiporiabannaensis* Y.C. Dai and *F.punctata* (P. Karst.) Murrill, retrieved from GenBank were used as the outgroup in the ITS+nLSU analysis (Fig. 2; Nie et al. 2017). Sequences of *Fasciodontiabrasiliensis* Yurchenko & Riebesehl and *F.bugellensis* (Ces.) Yurchenko, Riebesehl & Langer retrieved from GenBank were used as the outgroup in the ITS+nLSU analysis (Fig. 3; Yuan et al. 2024). Sequences of *Basidioradulummayi* Xue W. Wang & L.W. Zhou and *B.radula* (Fr.) Nobles retrieved from GenBank were used as the outgroup in the ITS+nLSU analysis (Fig. 4; Wang et al. 2023a; Dong et al. 2024).

Maximum Parsimony (MP), Maximum Likelihood (ML), and Bayesian Inference (BI) analyses were applied to the combined three datasets following a previous study (Dong et al. 2024). All characters were equally weighted and gaps were treated as missing data. Trees were inferred using the heuristic search option with TBR branch swapping and 1,000 random sequence additions. Max-trees were set to 5,000, branches of zero length were collapsed and all parsimonious trees were saved. Clade robustness was assessed using bootstrap (BT) analysis with 1,000 pseudo replicates (Felsenstein 1985). Descriptive tree statistics of tree length (TL), composite consistency index (CI), composite retention index (RI), composite rescaled consistency index (RC), and composite homoplasy index (HI) were calculated for each maximum parsimonious tree generated. The combined dataset was also analysed using Maximum Likelihood (ML) in RAxML-HPC2 through the CIPRES Science Gateway (Miller et al. 2012). Branch support (BS) for the ML analysis was determined by 1000 bootstrap pseudo replicates.

MrModeltest 2.3 (Nylander 2004) was used to determine the best-ﬁt evolution model for each dataset for the purposes of Bayesian inference (BI) which was performed using MrBayes 3.2.7a with a GTR+I+G model of DNA substitution and a gamma distribution rate variation across sites (Ronquist et al. 2012). A total of four Markov chains were run for two runs from random starting trees for 1 million generations for ITS+nLSU (Fig. 1); 0.8 million generations for ITS+nLSU (Figs 2, 3); and 4.4 million generations for ITS+nLSU (Fig. 4) with trees and parameters sampled every 1,000 generations. The ﬁrst quarter of all of the generations were discarded as burn-ins. A majority rule consensus tree was computed from the remaining trees. Branches were considered as significantly supported if they received a maximum likelihood bootstrap support value (BS) of ≥ 70%, a Maximum Parsimony bootstrap support value (BT) of ≥ 70% or a Bayesian posterior probability (BPP) of ≥ 0.95.

## ﻿Results

### ﻿Molecular phylogeny

The ITS+nLSU dataset (Fig. 1) comprised sequences from 102 fungal specimens representing 63 taxa. The dataset had an aligned length of 2,441 characters, of which 1,233 characters were constant, 204 were variable and parsimony-uninformative and 1,004 (50%) were parsimony-informative. Maximum Parsimony analysis yielded 3 equally parsimonious trees (TL = 7,192, CI = 0.3420, HI = 0.6803, RI = 0.7080 and RC = 0.2263). The best model of nucleotide evolution for the ITS+nLSU dataset estimated and applied in the Bayesian analysis was found to be GTR+I+G. Bayesian analysis and ML analysis resulted in a similar topology as in the MP analysis. The Bayesian analysis had an average standard deviation of split frequencies = 0.008504 (BI). The phylogram based on the ITS+nLSU rDNA gene regions (Fig. 1) included six families within the Hymenochaetales, which were Hymenochaetaceae, Hyphodontiaceae, Peniophorellaceae, Schizoporaceae, Skvortzoviaceae and Tubulicrinaceae, in which the new species *Hymenochaetebannaensis* was nested in Hymenochaetaceae; two taxa *Peniophorellaalbohymenia* and *P.punctata* were nested in Peniophorellaceae; the species *Lyomycesasiaticus* was grouped in Schizoporaceae.

The ITS+nLSU dataset (Fig. 2) comprised sequences from 80 fungal specimens representing 71 taxa. The dataset had an aligned length of 2,285 characters, of which 1,479 characters were constant, 199 were variable and parsimony-uninformative and 607 (50%) were parsimony-informative. Maximum Parsimony analysis yielded 3 equally parsimonious trees (TL = 3,749, CI = 0.3420, HI = 0.6580, RI = 0.5462 and RC = 0.1868). The best model of nucleotide evolution for the ITS+nLSU dataset estimated and applied in the Bayesian analysis was found to be GTR+I+G. Bayesian analysis and ML analysis resulted in a similar topology as in the MP analysis. The Bayesian analysis had an average standard deviation of split frequencies = 0.001385 (BI) and the effective sample size (ESS) across the two runs is double the average ESS (avg. ESS) = 365. The phylogram based on the ITS+nLSU rDNA gene regions (Fig. 1) highlighted that the new species *Hymenochaetebannaensis* was sister to *H.muroiana* I. Hino & Katum. with strong supports values (100/100/1.00), and then was closely nested with *H.colliculosa* (Sacc.) Parmasto, *H.globispora* G.A. Escobar and *H.megaspora* S.H. He & Hai J. Li.

The ITS+nLSU dataset (Fig. 3) comprised sequences from 51 fungal specimens representing 32 taxa. The dataset had an aligned length of 2,066 characters, of which 1,366 characters were constant, 177 were variable and parsimony-uninformative and 523 (50%) were parsimony-informative. Maximum Parsimony analysis yielded 3 equally parsimonious trees (TL = 2,358, CI = 0.4440, HI = 0.5560, RI = 0.6763 and RC = 0.3003). The best model of nucleotide evolution for the ITS+nLSU dataset estimated and applied in the Bayesian analysis was found to be GTR+I+G. Bayesian analysis and ML analysis resulted in a similar topology as in the MP analysis. The Bayesian analysis had an average standard deviation of split frequencies = 0.006833 (BI) and the effective sample size (ESS) across the two runs is double the average ESS (avg. ESS) = 306. The phylogram based on the ITS+nLSU rDNA gene regions (Fig. 3) indicated that the new species *Lyomycesasiaticus* was clustered with *L.crustosus* (Pers.) P. Karst., *L.ochraceoalbus* C.L. Zhao, *L.hengduanensis* Q. Yuan & C.L. Zhao and *L.zhaotongensis* Q. Yuan & C.L. Zhao.

The ITS+nLSU dataset (Fig. 4) comprised sequences from 36 fungal specimens representing 212 taxa. The dataset had an aligned length of 2,016 characters, of which 1,497 characters were constant, 72 were variable and parsimony-uninformative and 447 (50%) were parsimony-informative. Maximum Parsimony analysis yielded 3 equally parsimonious trees (TL = 1,235, CI = 0.6024, HI = 0.3976, RI = 0.7813 and RC = 0.4707). The best model of nucleotide evolution for the ITS+nLSU dataset estimated and applied in the Bayesian analysis was found to be TIM3+I+G. Bayesian analysis and ML analysis resulted in a similar topology as in the MP analysis. The Bayesian analysis had an average standard deviation of split frequencies = 0.009797 (BI) and the effective sample size (ESS) across the two runs is double the average ESS (avg. ESS) = 563. The phylogram based on the ITS+nLSU rDNA gene regions (Fig. 3) revealed that the new species *Peniophorellaalbohymenia* was grouped with two taxa as *P.olivacea* J.H. Dong & C.L. Zhao and *P.subpraetermissa* (Sheng H. Wu) K.H. Larss. Furthermore, the species *P.punctata* Y.L. Deng & C.L. Zhao was the sister to *P.fissurata* C.L. Zhao with strong supports values (100/100/1.00).

### ﻿Taxonomy

#### ﻿ Hymenochaete bannaensis

Taxon classificationFungiHymenochaetalesHymenochaetacea

Y.L. Deng & C.L. Zhao sp. nov.

72878E4B-89CC-5808-864A-64EC12E407A2

857317

Figs 5 , 6 , 7

##### Holotype.

China • Yunnan Province, Xishuangbanna, Wild elephant Valley, GPS coordinates: 22°17'N, 100°85'E, altitude 900 m asl., on the dead bamboo, leg. C.L. Zhao, 25 January 2024, CLZhao 35721 (SWFC).

##### Etymology.

*Bannaensis* (Lat.): referring to the locality (*banna*) of the type specimen.

##### Diagnosis.

*Hymenochaetebannaensis* is characterized by the flocculent basidiomata with cinnamon to yellowish brown to rust-brown hymenial surface, generative hyphae with simple septa and broadly ellipsoid to globose basidiospores.

##### Description.

***Basidiomata*** annual, resupinate, closely adnate, flocculent, without odor or taste when fresh, up to 7 cm long, 3.5 cm wide, and 200 μm thick. ***Hymenial surface*** smooth, cinnamon to yellowish brown when fresh, yellowish brown to rust-brown upon drying. Sterile margin yellowish brown, thinning out, up to 2 mm.

**Figure 5.**
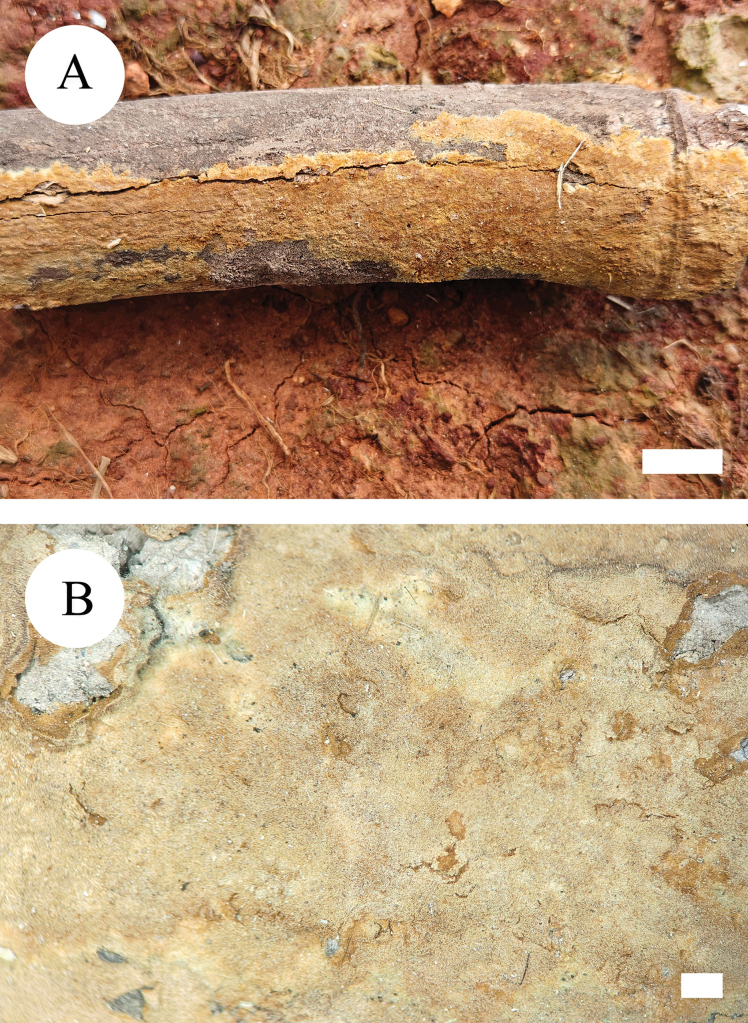
Basidiomata of *Hymenochaetebannaensis* (holotype, CLZhao 35721). Scale bars: 1 cm (**A**); 1 mm (**B**).

***Hyphal system*** monomitic; generative hyphae with simple-septa, brown, thick-walled, smooth, moderately branched, 2.4–2.9 µm in diameter, IKI–, CB–, tissues darkening in KOH; subhymenial hyphae densely covered by crystals.

**Figure 6.**
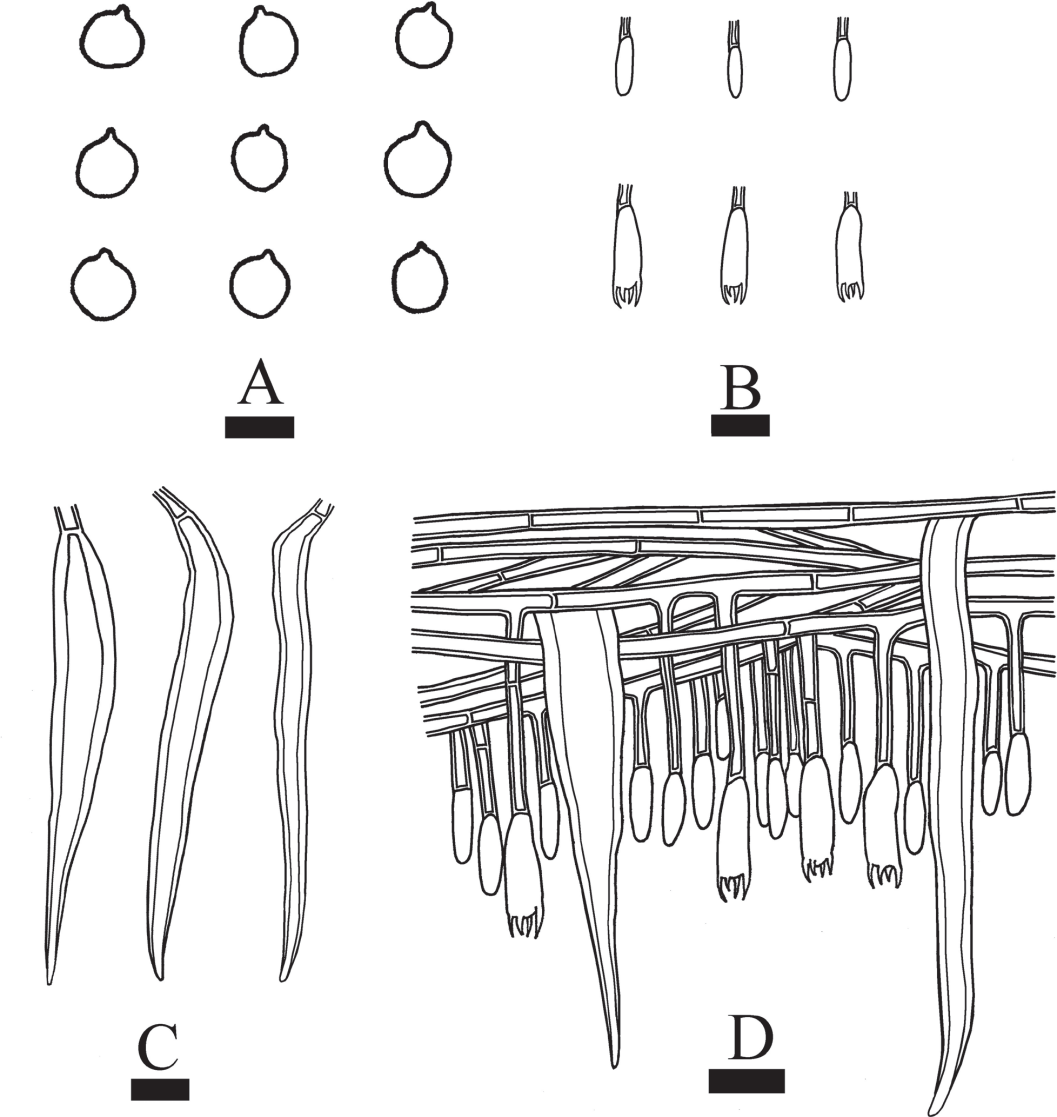
Microscopic structures of *Hymenochaetebannaensis* (holotype, CLZhao 35721) **A** basidiospores **B** basidia and basidioles **C** setae **D** a section of hymenium. Scale bars: 5 µm (**A**); 10 µm (**B–D**).

***Cystidia*** and ***cystidioles*** absent. ***Basidia*** subcylindrical, colorless, thin-walled, with four sterigmata and a basal simple septum, 18.5–23 × 3.5–4 µm; basidioles numerous, in shape similar to basidia but smaller. ***Setae*** abundant, distinctly thick-walled, subulate, with an acute tip, yellowish to reddish brown, 67–94 × 7–11 µm, projecting out of the hymenium up to 33–44.5 µm.

**Figure 7.**
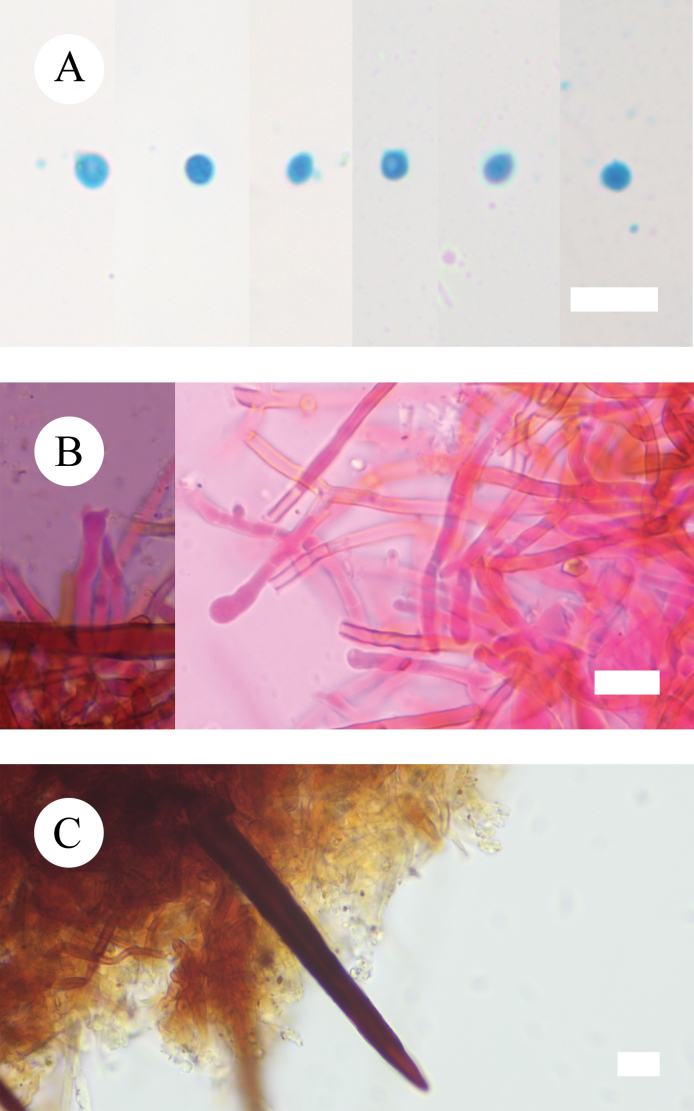
Sections of hymenium of *Hymenochaetebannaensis* (holotype, CLZhao 35721). Scale bars: 10 µm (**A–C**).

***Basidiospores*** broadly ellipsoid to globose, colorless, thin-walled, smooth, always filled with oil drop, CB–, (3.6–)3.7–4(–4.1) × (3.4–)3.5–3.9(–4) µm, L = 3.87 µm, W = 3.67 µm, Q = 1.05–1.06 (n = 60/2).

##### Another specimen (paratype) examined.

China • Yunnan Province, Xishuangbanna, Wild elephant Valley, GPS coordinates: 22°17'N, 100°85'E, altitude 900 m asl., on the fallen angiosperm branch, leg. C.L. Zhao, 25 January 2024, CLZhao 35884 (SWFC).

##### Notes.

Phylogenetically, *Hymenochaetebannaensis* is nested as sister to *H.muroiana* with strong supports (100/100/1.00) based on ITS+nLSU sequences (Figs 1, 2). Morphologically, *H.muroiana* is different from *H.bannaensis* by the brun suie basidiomata, smaller ellipsoid to subcylindrical basidia (8–10 × 3 µm) and narrower ovoid to ellipsoid basidiospores (3.5–4 × 2–2.6 µm; Léger 1998). *Hymenochaeteacerosa* S.H. He & Hai J. Li, *H.cystidiata* Parmasto and *H.sinensis* Y.C. Li & C.L. Zhao, are similar to *H.bannaensis* by thick-walled and acute setae. However, *H.acerosa* is distinguished from *H.bannaensis* by its effused and detachable basidiomata, longer setae (85–170 × 5–8 µm), wider clavate basidia (13–21 × 5–8 µm) and larger basidiospores (7–8.5 × 4.8–6 µm; He and Liu 2011). Furthermore, *H.cystidiata* differs from *H.bannaensis* by its longer setae (60–100 × 7–10 µm), and subcylindrical basidiospores (4.8–5.8 × 2.5–3.2 µm; Parmasto 2012). Moreover, *H.sinensis* differs from *H.bannaensis* by having ceraceous basidiomata, coconut brown to brownish black hymenial surface, shorter barrel-shaped basidia (10–12 × 3.1–3.8 µm) and longer ellipsoid to broadly ellipsoid basidiospores (4–5 × 2.5–3.5 µm; Li et al. 2024).

#### ﻿ Lyomyces asiaticus

Taxon classificationFungiCorticialesCorticiaceae

Y.L. Deng & C.L. Zhao sp. nov.

0650C8BE-09FF-5B7A-8DE6-0F72120B5737

857318

Figs 8 , 9 , 10

##### Holotype.

China • Yunnan Province, Xishuangbanna, Wild elephant Valley, GPS coordinates: 22°17'N, 100°85'E, altitude 900 m asl., on the dead bamboo, leg. C.L. Zhao, 25 January 2024, CLZhao 35719 (SWFC).

##### Etymology.

*Asiaticus* (Lat.): referring to the continent (Asia) where the species was found.

##### Diagnosis.

*Lyomycesasiaticus* is characterized by the membranaceous basidiomata with cream to pale-yellow hymenial surface with tuberculate, a monomitic hyphal system with clamped generative hyphae, and ellipsoid basidiospores 4.6–6.4 × 3–3.9 µm.

##### Description.

***Basidiomata*** annual, resupinate, closely adnate, membranaceous, without odor or taste when fresh, up to 5 cm long, 1.5 cm wide, and 100 μm thick. ***Hymenial surface*** tuberculate, thin, white to cream when fresh, cream upon drying. Sterile margin white to cream, thinning out, up to 2 mm.

***Hyphal system*** monomitic; generative hyphae with clamp connections, colorless, thin-walled, smooth, moderately branched, with some crystals, 2–2.5 µm in diameter, IKI–, CB–, tissues unchanged in KOH; subhymenial hyphae densely covered by crystals.

**Figure 8.**
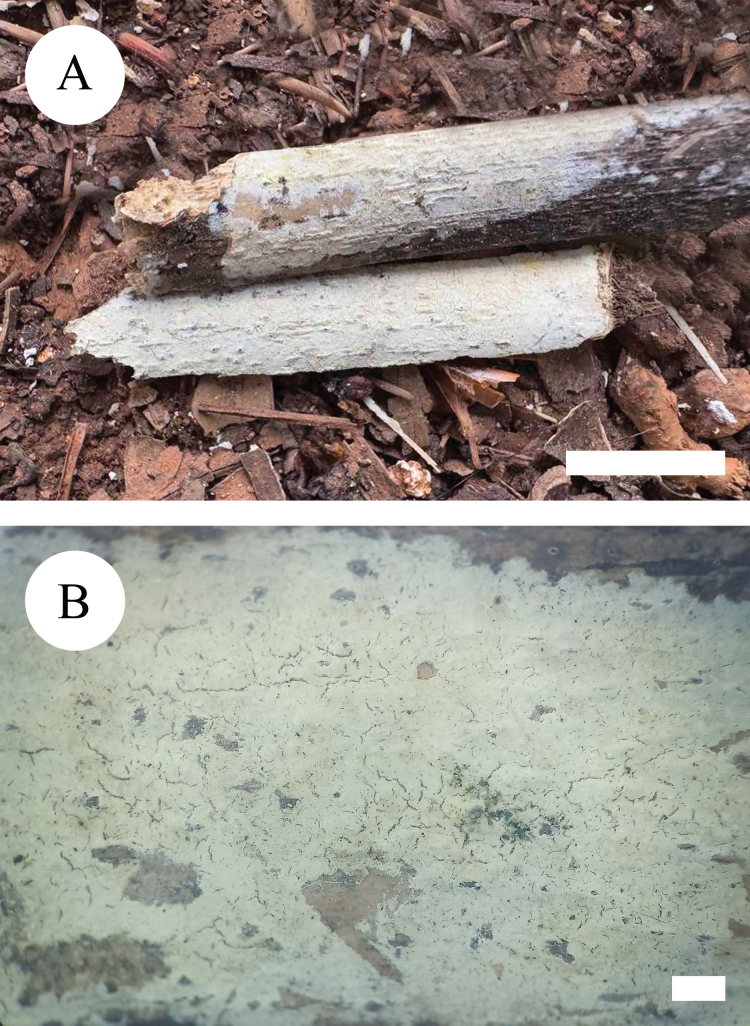
Basidiomata of *Lyomycesasiaticus* (holotype, CLZhao 35719). Scale bars: 1 cm (**A**); 1 mm (**B**)

***Hymenium*** three kinds of cystidia: (1) tapering, colorless, thin-walled, smooth, filled with refractive oil-like matter, 19.5–27 × 1.8–2.3 µm; (2) bottled, colorless, thin-walled, smooth, filled with refractive oil-like matter, 19.3–24.7 × 4.6–5.4 µm; (3) halocystidia, colorless, thin-walled, smooth, filled with refractive oil-like matter, 17–19 × 5.5–7 µm. ***Basidia*** cylindrical, with a basal clamp connection and four sterigmata, filled with refractive oil-like matter, 17–21 × 3–7 µm; basidioles numerous, in shape similar to basidia but smaller.

**Figure 9.**
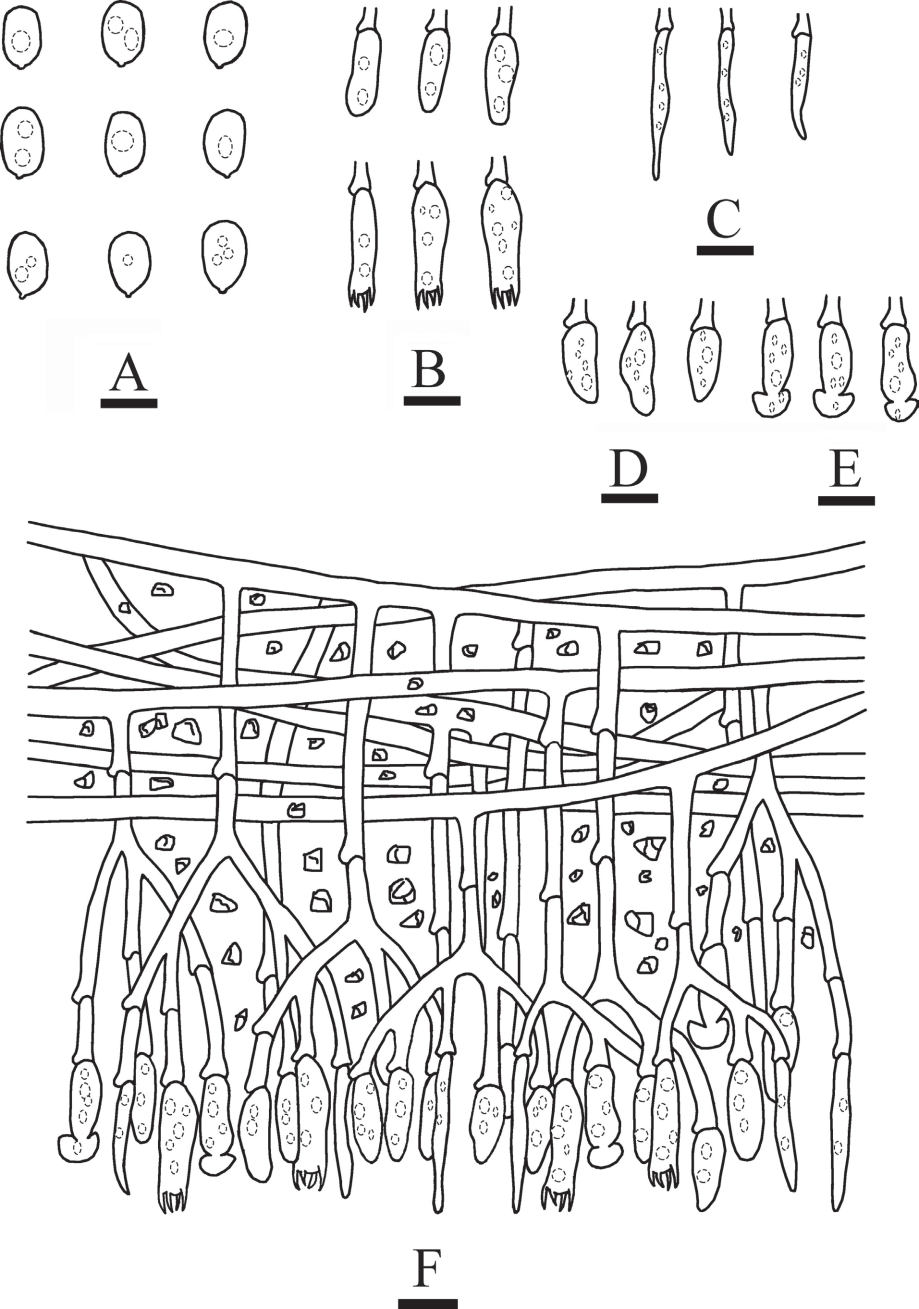
Microscopic structures of *Lyomycesasiaticus* (holotype, CLZhao 35719) **A** basidiospores **B** basidia and basidioles **C** tapering cystidia **D** bottled cystidia **E** halocystidia **F** a section of hymenium. Scale bars: 5μm (**A**); 10 μm (**B–F**).

***Basidiospores*** ellipsoid, colorless, thin-walled, smooth, always filled with oil drop, CB–, (4.2–)4.6–6.4(–6.5) × (2.4–)3–3.9(–4) µm, L = 5.63 µm, W = 3.36 µm, Q = 1.62–1.73 (n = 60/2).

##### Another specimen (paratype) examined.

China • Yunnan Province, Xishuangbanna, Wild elephant Valley, GPS coordinates: 22°17'N, 100°85'E, altitude 900 m asl., on the fallen angiosperm branch, leg. C.L. Zhao, 25 January 2024, CLZhao 35703 (SWFC).

**Figure 10.**
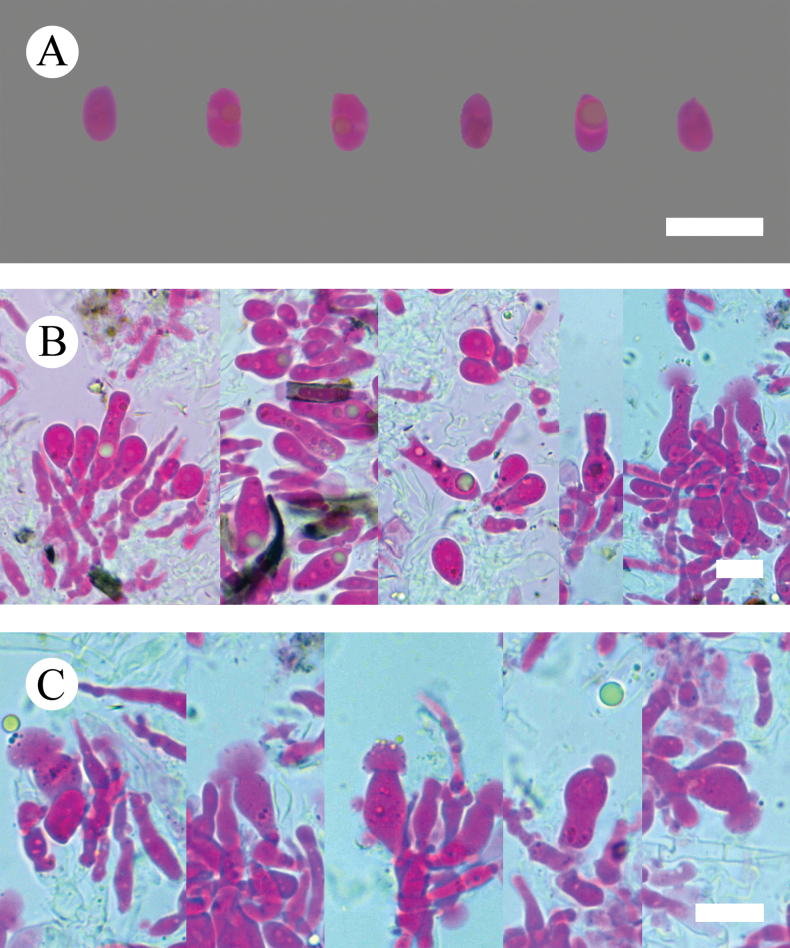
Sections of hymenium of *Lyomycesasiaticus* (holotype, CLZhao 35719). Scale bars: 10 µm (**A–C**).

##### Notes.

The phylogenetic tree of ITS+nLSU (Fig. 3) showed that the new species *Lyomycesasiaticus* was clustered with *L.crustosus* and *L.ochraceoalbus*. However, morphologically, *L.crustosus* can be delimited from *L.asiaticus* by having effused basidiocarp, clavate basidia (26 × 4.5 µm) and longer ellipsoidal to subcylindrical basidiospores (5–7.5 × 2.5–3 µm; Karsten 1881). *L.ochraceoalbus* differs from *L.asiaticus* in its smooth hymenial surface, lacking a cystidium and shorter clavate basidia (11–16.5 × 3.5–5 µm; Luo et al. 2021b). Morphologically, *Lyomycesasiaticus* reminds *L.albopulverulentus* C.L. Zhao, *L.cremeus*, *L.denudatus* Viner, *L.macrosporus* C.L. Zhao and *L.wuliangshanensis* C.L. Zhao by having the ellipsoid basidiospores. However, *L.albopulverulentus* can be separated from *L.asiaticus* by its pruinose hymenial surface and larger basidia (24.5–28.5 × 7–9 µm) and larger basidiospores (8–10.5 × 5.5–7 µm; Guan et al. 2023). *L.cremeus* is distinct from *L.asiaticus* by its smooth hymenial surface, present two types cystidia as capitate (20–40 × 3–5 µm) and tapering (18–35 × 3–4.5 µm) and thin-walled to slightly thick-walled basidiospores (Chen and Zhao 2020). In addition, *L.denudatus* is separated from *L.asiaticus* by the smooth hymenial surface, longer capitate cystidia (34.9–62 × 4–5.5 µm) and suburniform basidia (15–21.1 × 3.8–5.5 µm; Viner and Miettinen 2022). The species *L.macrosporus* differs from *L.asiaticus* due to its reticulate hymenial surface and longer basidia (22.2–38 × 4.5–7 µm) and larger basidiospores (6.7–8.9 × 4.4–5.4 µm; Chen and Zhao 2020). Moreover, *L.wuliangshanensis* can be delimited from *L.asiaticus* by its smooth to more or less tuberculate hymenial surface and two kinds of cystidia as capitate (22–37 × 3–6 µm) and tapering (21–35 × 4–6.5 µm; Chen and Zhao 2020).

#### ﻿ Peniophorella albohymenia

Taxon classificationFungiHymenochaetalesRickenellaceae

Y.L. Deng & C.L. Zhao sp. nov.

4C4257F1-7FE5-5508-82F6-62CDF1FAA5C2

857319

Figs 11 , 12 , 13

##### Holotype.

China • Yunnan Province, Zhaotong, Xiaocaoba Town, Wumengshan National Nature Reserve, GPS coordinates: 27°33'N, 103°27'E, altitude 2300 m asl., on fallen angiosperm branch, leg. C.L. Zhao, 19 September 2023, CLZhao 33187 (SWFC).

##### Etymology.

*Albohymenia* (Lat.): referring to “albus”, the distinctive white hymenium of the type specimen when fresh.

##### Diagnosis.

*Peniophorellaalbohymenia* is characterized by the membranaceous basidiomata with white hymenial surface, four types cystidia as stephanocyst, fusiform, cylindrical and capitate, and ellipsoid basidiospores measuring 9–10.9 × 4.5–5 µm.

##### Description.

***Basidiomata*** annual, resupinate, adnate, membranaceous, without odor or taste when fresh, up to 4.5 cm long, 1 cm wide, and 0.1 mm thick. ***Hymenial surface*** smooth, white when fresh and dry. Sterile margin distinctly, thin, white, up to 1 mm long.

**Figure 11.**
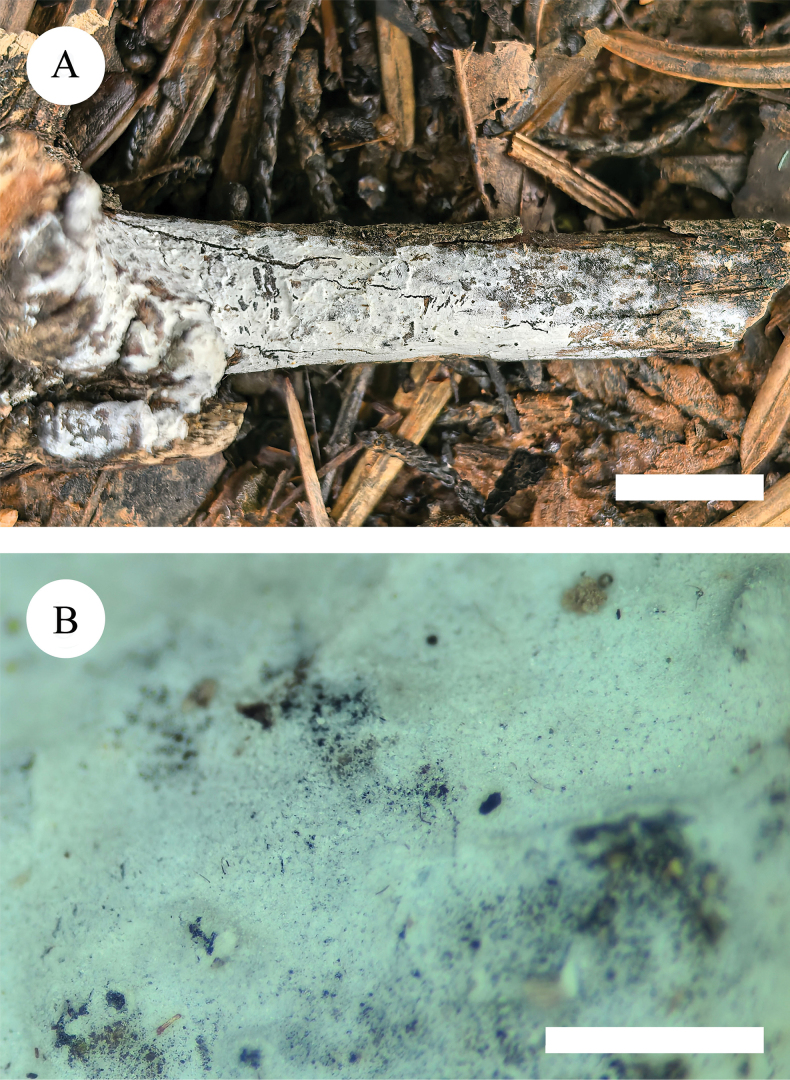
Basidiomata of *Peniophorellaalbohymenia* (holotype, CLZhao 33187) **A** basidiomata on the substrate **B** character hymenophore Scale bars: 1 cm (**A**); 1 mm (**B**).

***Hyphal system*** monomitic; generative hyphae with clamp connections, thin-walled, colorless, occasionally branched, 2.5–4 µm in diameter, IKI–, CB–, tissues unchanged in KOH.

**Figure 12.**
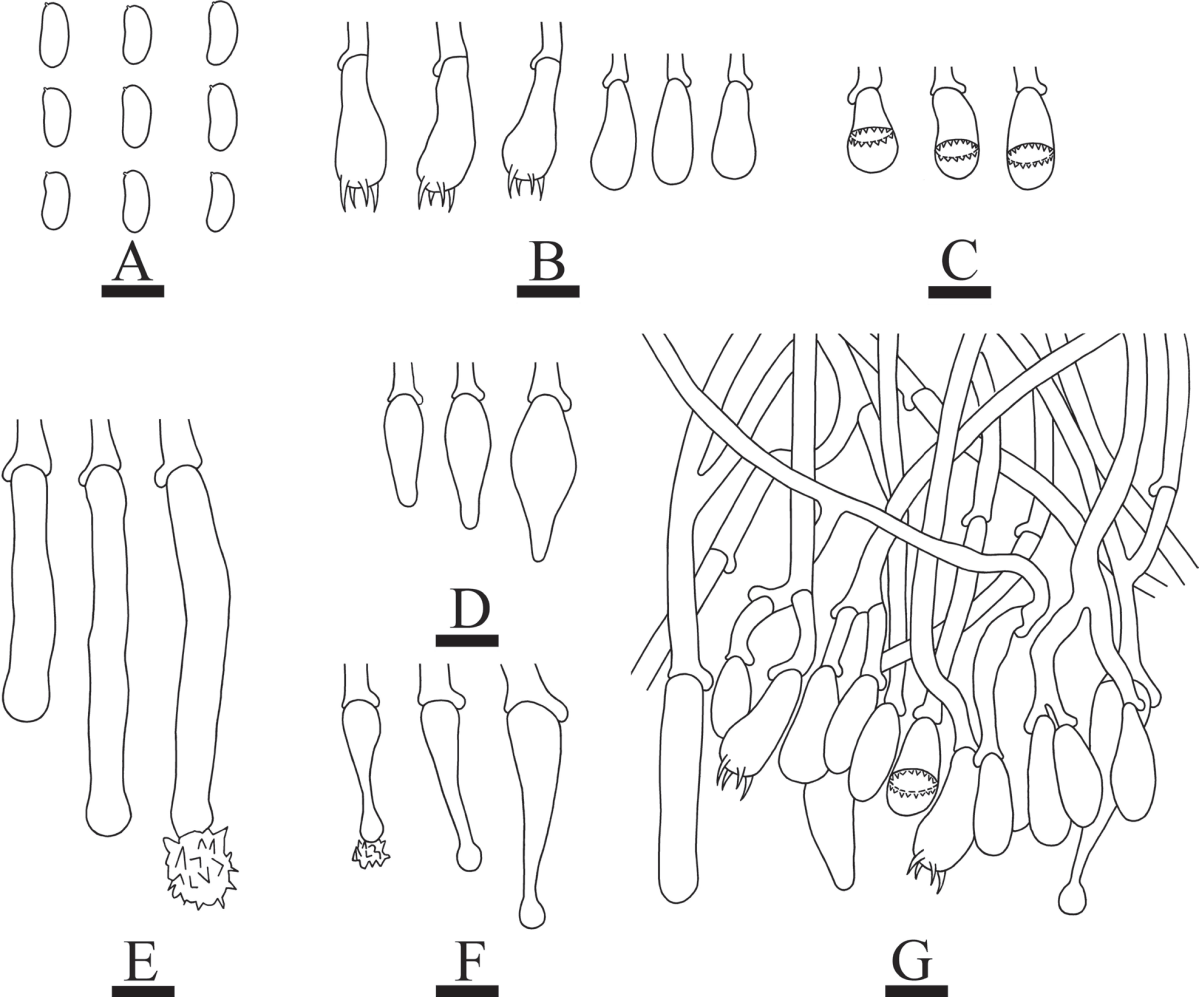
Microscopic structures of *Peniophorellaalbohymenia* (holotype, CLZhao 33187) **A** basidiospores **B** basidia and basidioles **C** stephanocyst **D** fusiform cystidia **E** cylindrical cystidia **F** capitate cystidia **G** a section of the hymenium. Scale bars: 10 µm (**A–G**).

***Cystidia*** of four types: (1) stephanocyst, thin-walled, 9–18 × 7–8 µm; (2) fusiform cystidia, smooth, thin-walled, 29–31 × 7–9 µm; (3) cylindrical cystidia, thin-walled, slightly constricted at the neck, the apical part encrusted with asteroid, 27–64 × 5–11 µm; (4) capitate cystidia, thin-walled, the apical part encrusted with asteroid, 22–47 × 5–12 µm. ***Basidia*** clavate, with four sterigmata and a basal clamp connection, colorless, thin-walled, 20–28 × 7.5–9 µm; basidioles in shape similar to basidia, but slightly smaller.

**Figure 13.**
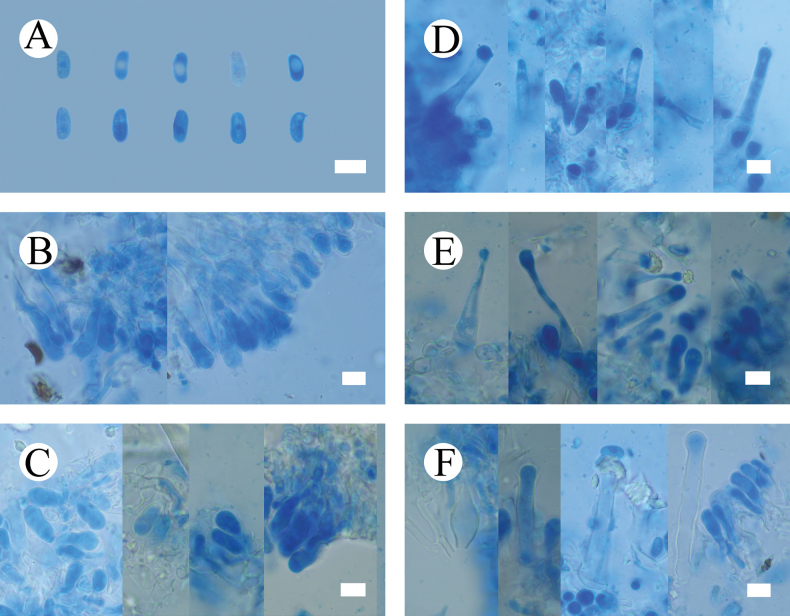
Sections of hymenium of *Peniophorellaalbohymenia* (holotype, CLZhao 33187). Scale bars: 10 µm (**A–F**).

***Basidiospores*** ellipsoid, colorless, thin-walled, IKI–, CB–, (8.5–)9–10.9(–11.2) × (4.4–)4.5–5(–5.5) µm, L = 9.87 µm, W = 4.93 µm, Q = 1.99–2.02 (n = 60/2).

##### Another specimen (paratype) examined.

China • Yunnan Province, Zhaotong, Xiaocaoba Town, Wumengshan National Nature Reserve, GPS coordinates: 27°33'N, 103°27'E, altitude 2300 m asl., on fallen angiosperm branch, leg. C.L. Zhao, 19 September 2023, CLZhao 33257 (SWFC).

##### Notes.

Our results indicate that the new species *P.albohymenia* was placed within *Peniophorella* inferring from the dataset of ITS+nLSU (Figs 1, 4). Morphologically, the species *P.albohymenia* is similar to *P.daweishanensis* J.H. Dong & C.L. Zhao and *P.yunnanensis* C.L. Zhao by having capitate cystidia, but *P.daweishanensis* is distinct from *P.albohymenia* by its buff to slightly yellowish, reticulate hymenial surface and longer basidia (31.5–38 × 7–9 µm; Dong et al. 2024). *P.yunnanensis* differs from *P.albohymenia* due to its grandinioid hymenial surface (Guan et al. 2020).

#### ﻿ Peniophorella punctata

Taxon classificationFungiHymenochaetalesRickenellaceae

Y.L. Deng & C.L. Zhao sp. nov.

995B8CD2-FA3D-538A-AAAD-C728CB72AC0D

857320

Figs 14 , 15 , 16

##### Holotype.

China • Yunnan Province, Zhaotong, Xiaocaoba Town, Wumengshan National Nature Reserve, GPS coordinates: 27°33'N, 103°27'E, altitude 2300 m asl., on fallen angiosperm branch, leg. C.L. Zhao, 21 September 2023, CLZhao 33732 (SWFC).

##### Etymology.

*Punctata* (Lat.): referring to the punctate basidioma of the type specimen.

##### Diagnosis.

*Peniophorellapunctata* is characterized by the membranaceous, punctate basidiomata with white to pale yellow hymenial surface, fusiform cystidia, and allantoid basidiospores measuring 9.5–12.5 × 4–4.8 µm.

**Figure 14.**
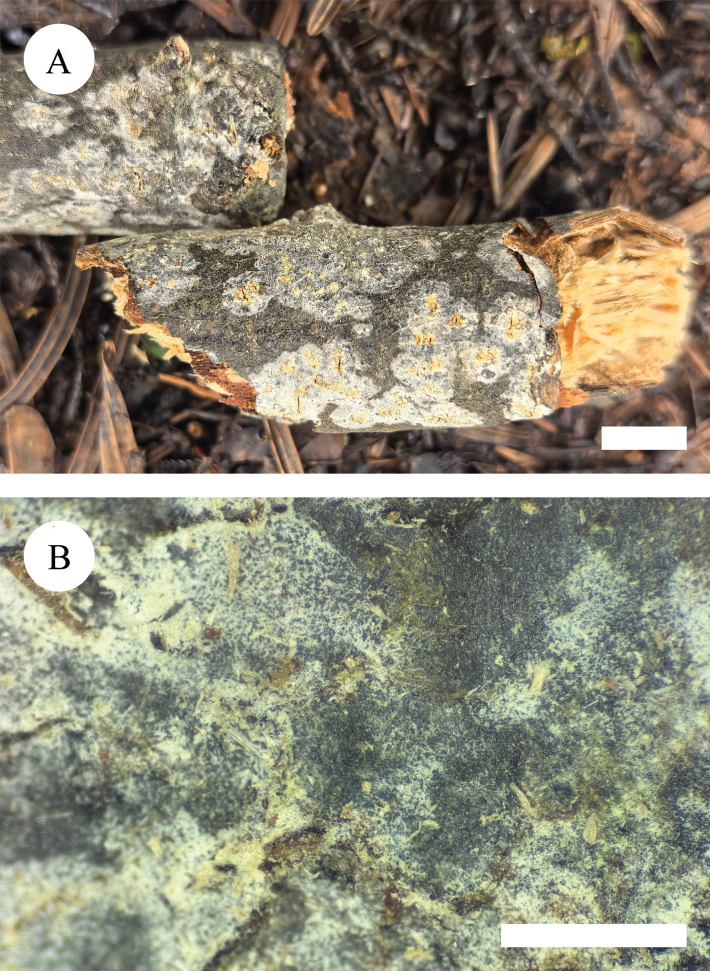
Basidiomata of *Peniophorellapunctata* (holotype, CLZhao 33732) **A** basidiomata on the substrate **B** character hymenophore. Scale bars: 1 cm (**A**); 1 mm (**B**).

##### Description.

***Basidiomata*** annual, resupinate, adnate, membranaceous, punctate, without odor or taste when fresh, up to 4 cm long, 3 cm wide, and 0.1 mm thick. ***Hymenial surface*** smooth, white to pale yellow when fresh, turning to aurantiacus to avellaneus upon drying. Sterile margin distinctly, thin, white, up to 2 mm long.

**Figure 15.**
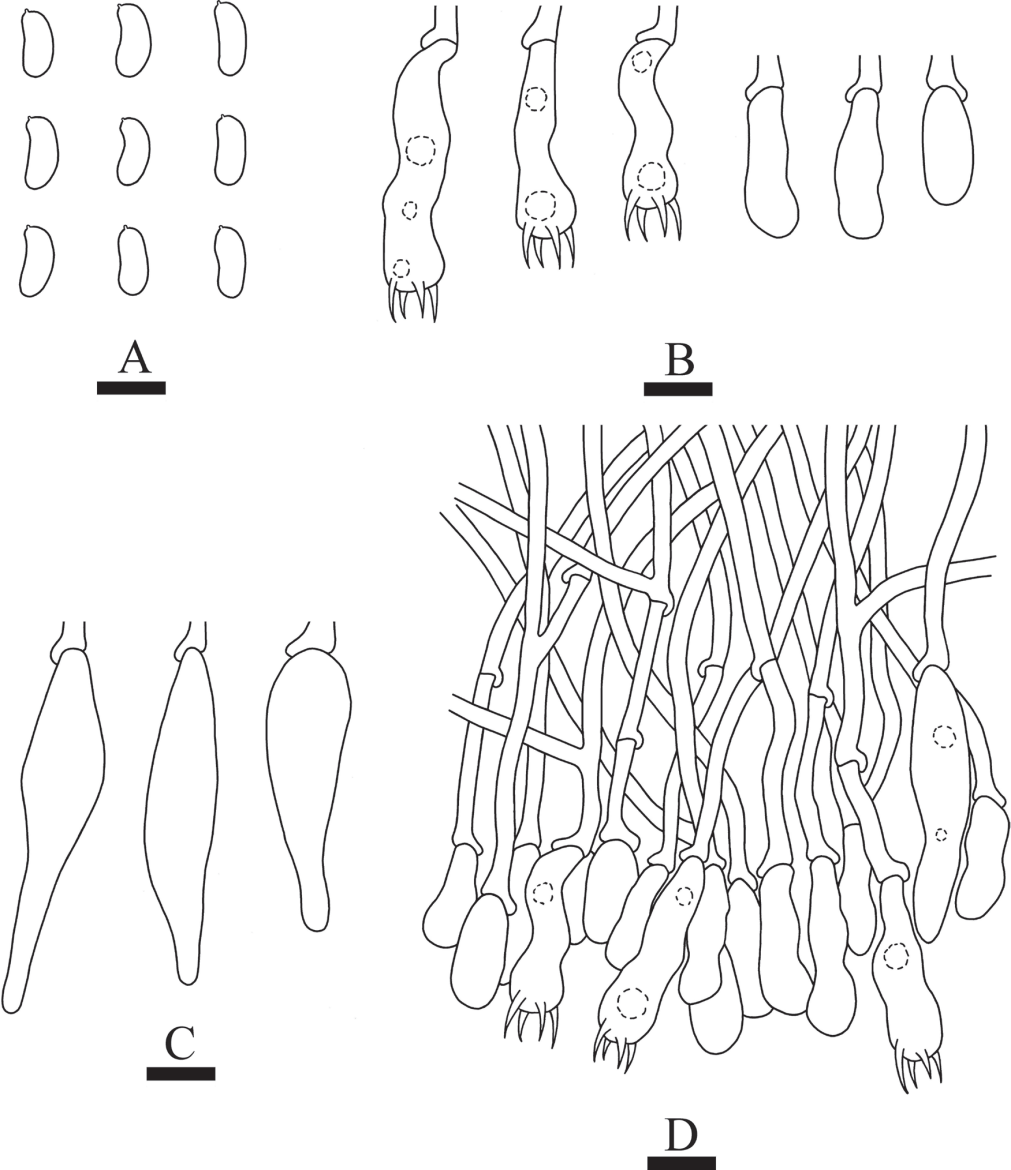
Microscopic structures of *Peniophorellapunctata* (holotype, CLZhao 33732) **A** basidiospores **B** basidia and basidioles **C** fusiform cystidia **D** a section of the hymenium. Scale bars: 10 µm (**A–D**).

***Hyphal system*** monomitic, generative hyphae with clamp connections, thin-walled, colorless, occasionally branched, 2.9–4.6 µm in diameter, IKI–, CB–, tissues unchanged in KOH.

**Figure 16.**
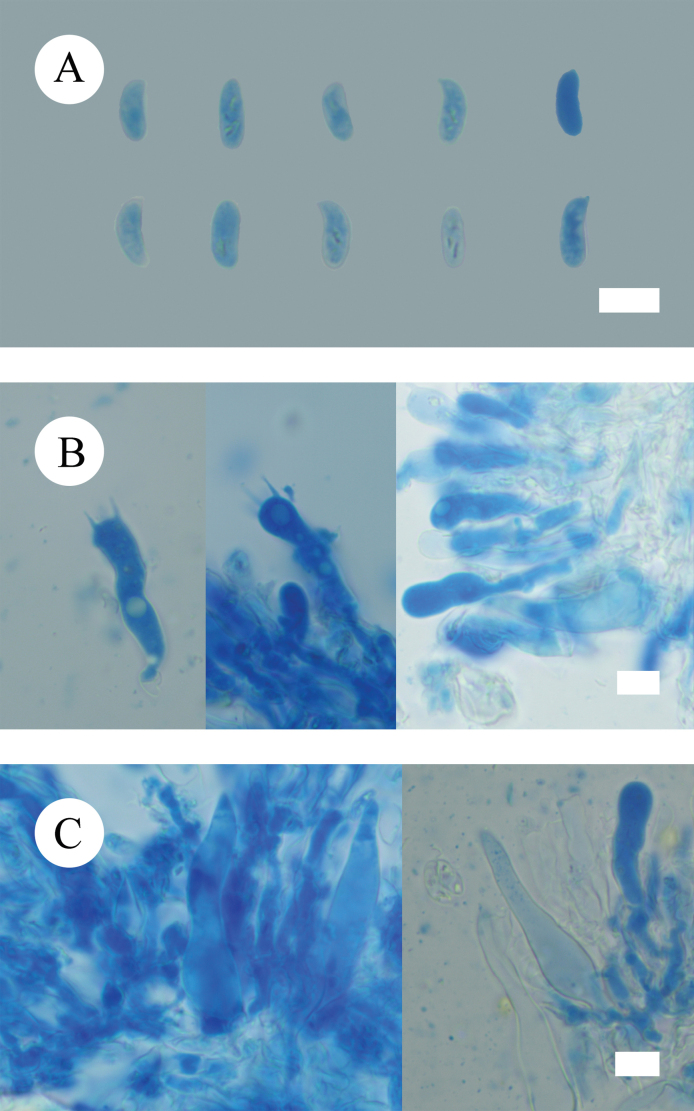
Sections of hymenium of *Peniophorellapunctata* (holotype, CLZhao 33732). Scale bars: 10 µm (**A–C**).

***Cystidia*** fusiform, slightly constricted at the neck and forms a long beak, thin-walled, 31–57 × 9–13.5 µm. ***Basidia*** clavate, slightly constricted in the middle, with four sterigmata and a basal clamp connection, colorless, thin-walled, 26.3–39.8 × 8.1–9.5 µm; basidioles in shape similar to basidia, but slightly smaller.

***Basidiospores*** cylindrical to allantoid, colorless, thin-walled, IKI–, CB–, (9.1–)9.5–12.5(–12.9) × (3.7–)3.9–4.8(–5.5) µm, L = 10.89 µm, W = 4.30 µm, Q = 2.53–2.54 (n = 60/2).

##### Another specimen (paratype) examined.

China • Yunnan Province, Zhaotong, Xiaocaoba Town, Wumengshan National Nature Reserve, GPS coordinates: 27°33'N, 103°27'E, altitude 2300 m asl., on fallen angiosperm branch, leg. C.L. Zhao, 21 September 2023, CLZhao 33720 (SWFC).

##### Notes.

The phylogenetic analysis indicates that species *P.punctata* was placed within *Peniophorella* inferring from the dataset of ITS+nLSU. Morphologically, *P.allantospora* (Sheng H. Wu) K.H. Larss., *P.capitulata* (Boidin & Gilles) K.H. Larss., *P.flagellata* (G. Cunn.) K.H. Larss., *P.pallida* (Bres.) K.H. Larss. and *P.praetermissa* (P. Karst.) K.H. Larss. are similar to *P.punctata* based on the smooth hymenophore and allantoid basidiospores. However, *P.allantospora* differs from *P.punctata* by its shorter subutriformia to subclavata basidia (17–27 × 7.0–8.5 µm; Larsson 2007b). *P.capitulata* in its presence of subcylindrica leptocystidia (25–50 × 4–7 µm; Larsson 2007b). *P.flagellata* is distinct from *P.punctata* by its cream hymenial surface, obovate or subglobose stephanocysts, narrower basidia (28–40 × 7–8 µm) and shorter basidiospores (8–9 × 5–6 µm; Larsson 2007b). *P.pallida* can be delimited from *P.punctata* by its whitish to ochraceous hymenial surface and longer fusiform cystidia (50–70 × 6–8 µm) and smaller basidia (20–25 × 5–6 µm; Larsson 2007b). *P.praetermissa* can be separated from *P.punctata* by presence of three types of cystidia as fusiform gloeocystidia (50–100 × 8–12 µm), cylindrical leptocystidia (20–80 × 6–8 µm) and stephanocysts bowl-shaped, and narrower basidia (20–30 × 6–7 µm; Larsson 2007b).

## ﻿Discussion

Many recently described wood-inhabiting fungal taxa have been reported worldwide, including in the genera *Hymenochaete*, *Lyomyces* and *Peniophorella* (Chen et al. 2017; Kan et al. 2017a, b; Pacheco et al. 2018; Miettinen et al. 2019; Viner and Miettinen 2022; Dong et al. 2024). The Xishuangbanna and Zhaotong are situated in the southwest of China; these areas become focal points for fungal diversity in China due to their complex topography and diverse ecosystems. In our study, four new species were found from Xishuangbanna and Zhaotong, Yunnan Province, China.

The family Hymenochaetaceae is characterized by annual to perennial, brownish basidiomata with a xanthochroic reaction in KOH, poroid or corticioid hymenophore, generative hyphae without clamp connections and setal elements present or absent (Wu et al. 2022a, b). Some forest pathogens species are mainly located in the genus *Coniferiporia* L.W. Zhou & Y.C. Dai, *Onnia* P. Karst. and *Pyrrhoderma* Imazeki (Zhou et al. 2016; Ji et al. 2017). Furthermore, *Inonotusobliquus* (Fr.) Pilát and *Sanghuangporussanghuang* (Sheng H. Wu, T. Hatt. & Y.C. Dai) Sheng H. Wu, L.W. Zhou & Y.C. Dai have therapeutic properties (Wu et al. 2019b; Lv et al. 2023; Zhang et al. 2023a; Ma et al. 2024). Hymenochaetaceae is typed with the genus *Hymenochaete* Lév., which was erected in 1846 and typified by *H.rubiginosa* (Dicks.) Lév. The genus *Hymenochaete* can be identified by the annual to perennial, resupinate, effused-reflexed to pileate basidiomata with smooth, lamellate, tuberculate, poroid or hydnoid hymenophore, hyphal system monomitic or dimitic, presence of setae, and colorless, thin-walled, narrowly cylindrical to globose basidiospores (Léger 1998; Parmasto 2001; He and Dai 2012). There are some characteristics that can be used to circumscribe the genus, such as brown basidiomata darkening in potassium hydroxide, simple-septate generative hyphae, brown setae, and causing a white rot (Léger 1998; Parmasto 2001). In the present study, the new species *H.bannaensis* is distinguished by flocculent basidiomata with cinnamon to yellowish brown to rust-brown hymenial surface, generative hyphae with simple septa and broadly ellipsoid to globose basidiospores. Phylogenetically, *H.bannaensis* was close to *H.colliculosa*, *H.globispora*, and *H.megaspora*. However, morphologically, *H.colliculosa* is distinguished from *H.bannaensis* by its brown hymenial surface with tuberculate and larger ellipsoid basidiospores measuring 6–7.5 × 4–4.5 µm (Léger 1998). Furthermore, *H.globispora* differs from *H.bannaensis* by its deep reddish-brown hymenial surface with granular, longer setae (150 µm) with crystal, wider basidia (15–18 × 4.5–5.5 µm) and globular basidiospores (4 × 5.5 µm; Léger 1990). Moreover, *H.megaspora* is distinct from *H.bannaensis* by having effused or effused-reflexed with slightly elevated margins basidiomata (150–600 µm thick), grayish brown to dark gray, silky, tomentose, concentrically sulcate and zonate pileal surface, smooth or tuberculate hymenophore with pale mouse-gray to vinaceous gray, longer setae (90–120 × 8–13 µm), larger clavate basidia (25–32 × 5–8 µm) and larger, broadly ellipsoid basidiospores measuring 7.5–10 × 5–7 µm (He and Liu 2011).

The genus *Lyomyces* P. Karst. (Schizoporaceae) was established by Karsten (1881) with *L.sambuci* (Pers.) P. Karst. as the type species. It is characterized by the resupinate-to-effused basidiomata with a smooth to odontioid hymenophore, a monomitic hyphal system with generative hyphae bearing clamp connections, the presence of several types of cystidia and with smooth, thin- to slightly thick-walled basidiospores (Karsten 1881; Bernicchia and Gorjón 2010). Previously, 77 specific and infraspecific names were registered in the Index Fungorum (http://www.indexfungorum.org; accessed on 24 March 2025), of which approximately 65 species of *Lyomyces* are currently accepted (Cunningham 1963; Dai 2011; Gafforov et al. 2017; Luo et al. 2021a; Yuan et al. 2024; Dai et al. 2025). In this study, the new species *Lyomycesasiaticus* is characterized by the membranaceous basidiomata with white to cream hymenial surface with tuberculate, a monomitic hyphal system with clamped generative hyphae and ellipsoid basidiospores. The phylogenetic tree of ITS+nLSU (Figs 1, 3) showed that *L.asiaticus* was grouped closely with *L.hengduanensis* and *L.zhaotongensis*. However, morphologically, *L.hengduanensis* is distinct from *L.asiaticus* by having pruinose hymenial surface, fusoid cystidia (17.5–25 × 3–4 µm) and subclavate cystidia (16–23 × 3–4.5 µm), and smaller clavate basidia (10.5–14 × 3.5–5 µm; Yuan et al. 2024). *L.zhaotongensis* is distinguishable from *L.asiaticus* by its farinaceous basidiomata, fusoid cystidia (16–20.5 × 2.5–3.5 µm), smaller clavate basidia (14–16.5 × 2.5–3.5 µm) and smaller broadly ellipsoid basidiospores measuring 2.6–3.5 × 2.5–3 µm (Yuan et al. 2024).

The family Peniophorellaceae L.W. Zhou et al. was established by Wang et al. (2023a), to include the genus *Peniophorella* P. Karst, typed with *P.pubera* (Fr.) P. Karst. Species of the family are characterized by annual, resupinate, effused, adnate, thin, ceraceous basidiomata with smooth to tuberculate, white to yellowish hymenophore, a monomitic hyphal system with clamped generative hyphae thin-walled, and colorless, the presence of different kinds of cystidia, metuloids, gloeocystidia or leptocystidia, echinulate cells usually present, narrowly to broadly clavate basidia with four sterigmata, and hyaline, thin-walled, smooth, ellipsoid, cylindrical or allantoid, inamyloid, acyanophilous basidiospores with oily contents (Wang et al. 2023a). So far, 37 species have been accepted in the genus worldwide (Prasher 2015; Guan et al. 2020; Xu et al. 2020; Dong et al. 2024; Liu et al. 2024). In the present study, two new species have been identified; *P.albohymenia* is delimited by its membranaceous basidiomata with white hymenial surface, four types cystidia as stephanocyst, fusiform, cylindrical and capitate, and ellipsoid basidiospores. Furthermore, *P.punctata* is unique in the membranaceous, punctate basidiomata with white to pale yellow hymenial surface, fusiform cystidia, and allantoid basidiospores. In our phylogenies (Figs 1, 4), *P.albohymenia* was grouped with *P.olivacea* and *P.subpraetermissa* with strong supports. Morphologically, *P.olivacea* can be separated from *P.albohymenia* by having the ceraceous to farinaceous basidiomata, grayish olivaceous hymenial surface, two types cystidia as fusiform (28.5–39 × 8.5–11 µm) and halocystidia (42–50 × 8–9 µm), smaller basidia (17.5–20 × 6–7 µm) and smaller basidiospores (6.5–7.5 × 3.2–4.5 µm; Dong et al. 2024). *P.subpraetermissa* is distinct from *P.albohymenia* by its longer subclavata basidia (20–35 × 6–8 µm) and smaller basidiospores (6.2–7.5 × 4–5 µm; Wu 1997). Furthermore, *P.punctata* was the sister to *P.fissurata* with strong supports. However, morphologically, the latter having the ceraceous to corneus basidiomata, smooth to tuberculate and white to cream hymenial surface and larger fusiform cystidia (28–65 × 6.5–15 µm; Guan et al. 2020).

The wood-inhabiting fungi are an extensively studied group of Basidiomycota, and the taxa of Hymenochaetales play a core group in the wood-inhabiting fungi (Dai 2012; Ryvarden and Melo 2014; Liu et al. 2021; Zhou et al. 2022; Deng et al. 2024; Wang et al. 2024), but the species of Hymenochaetales diversity is still not well-known in China, especially in subtropical and tropical areas, and many recently described taxa of this ecologically important Hymenochaetales group were reported from China (Zhao et al. 2014, 2015, 2016; Chen et al. 2020; Guan et al. 2021; Yu et al. 2021; Dong et al. 2024). In the present study, four new species within the order Hymenochaetales are described, based on both morphological and molecular phylogenetic analyses, and are also from the subtropics. This study enriches the wood-inhabiting fungal diversity in China.

## Supplementary Material

XML Treatment for Hymenochaete bannaensis

XML Treatment for Lyomyces asiaticus

XML Treatment for Peniophorella albohymenia

XML Treatment for Peniophorella punctata

## ﻿Additional information

### Author ORCIDs

Yinglian Deng https://orcid.org/0000-0002-8220-508X

Meng Chen https://orcid.org/0009-0005-9916-8704

Linfeng Liu https://orcid.org/0009-0003-4058-301X

Sicheng Zhang https://orcid.org/0009-0003-6287-9823

Haisheng Yuan https://orcid.org/0000-0001-7056-140X

Changlin Zhao https://orcid.org/0000-0002-8668-1075

### Data availability

All of the data that support the findings of this study are available in the main text.
